# Semaglutide and Panic Attacks: Active‐Comparator Disproportionality Analysis, Temporal Assessment of Notoriety Bias and Evidence Triangulation in Post‐Marketing Pharmacovigilance

**DOI:** 10.1002/pds.70517

**Published:** 2026-10-27

**Authors:** Ben Melamed, Mahak Parsi‐Zahabi, Mohammad Ali Omrani, Bala Swetha Baskaran, Lujain Ez Eddin, Nazgol Kermanshahi, Niaz Chalabianloo, Flory Tsobo Muanda

**Affiliations:** ^1^ Department of Physiology and Pharmacology Western University London Ontario Canada; ^2^ ICES Western, Kidney, Dialysis & Transplantation Research Program London Ontario Canada; ^3^ Department of Epidemiology and Biostatistics Western University London Ontario Canada; ^4^ London Health Sciences Centre, Lawson Health Research Institute London Ontario Canada

**Keywords:** confounding by indication, FAERS, GLP‐1 receptor agonists, notoriety bias, panic attack, pharmacovigilance, semaglutide

## Abstract

**Purpose:**

To evaluate how confounding by indication and notoriety bias shape the apparent semaglutide–panic attack signal in the FDA Adverse Event Reporting System (FAERS) and to interpret this signal using an evidence‐triangulation framework integrating randomized and observational studies.

**Methods:**

We conducted an active‐comparator restricted disproportionality analysis of FAERS (2018Q1–2024Q4), including individual case safety reports in which semaglutide, dulaglutide or empagliflozin was the primary suspect drug. Panic attack was identified using the MedDRA preferred term. Crude and adjusted reporting odds ratios (RORs, aRORs) with 95% confidence intervals (CIs) were estimated using logistic regression, adjusting for age, sex and indication (Type 2 diabetes, obesity/weight management). Notoriety bias was assessed using prespecified temporal analyses (analyses restricted to reports received before July 1, 2023 and a drug‐by‐period comparison of 2018–2022 with 2023–2024) and reporting proportions per 10 000 reports. A sensitivity analysis used all other primary‐suspect drugs in FAERS as the reference set. In parallel, we conducted a systematic review (MEDLINE, Embase, CENTRAL to November 24, 2025) of randomized trials, cohort and cross‐sectional studies, pharmacovigilance analyses and case reports reporting panic attacks or acute anxiety outcomes with semaglutide.

**Results:**

Among 33 267 semaglutide, 54 282 dulaglutide and 25 380 empagliflozin reports, panic attacks were recorded in 87 (0.26%), 28 (0.05%) and 13 (0.05%) cases, respectively. Crude RORs were 5.08 (95% CI 3.32–7.78) versus dulaglutide and 5.12 (95% CI 2.86–9.17) versus empagliflozin, attenuating after adjustment to 2.82 (95% CI 1.74–4.56) and 2.75 (95% CI 1.41–5.36). In the pre‐warning period crude RORs were 2.24 (95% CI 1.23–4.07) and 2.15 (95% CI 1.00–4.58), attenuating to 1.38 (95% CI 0.69–2.75) and 1.14 (95% CI 0.48–2.68) after adjustment. Panic‐attack reports per 10 000 semaglutide reports rose from 6.7 in 2018–2022 to 42.0 in 2023–2024 (drug‐by‐period interaction *p* = 0.007). Against all other primary‐suspect drugs, the aROR was 1.48 (95% CI 1.18–1.87) overall and 0.76 (95% CI 0.48–1.18) before July 1, 2023. Across 12 studies, randomized trials and active‐comparator observational cohorts did not show a consistent increase in panic attacks or anxiety‐related outcomes.

**Conclusions:**

Crude FAERS analyses suggested a semaglutide–panic attack signal that attenuated after adjustment was no longer significant before the July 2023 regulatory communication and rose sharply thereafter in a pattern consistent with notoriety bias. Triangulation with randomized trials and active‐comparator observational studies does not support a consistent pharmacological association. Disproportionality analyses are hypothesis‐generating and cannot estimate incidence or establish causality. This case study illustrates how active‐comparator design, temporal assessment of notoriety bias and evidence triangulation can improve the interpretation of post‐marketing safety signals.

## Key Points

- Crude FAERS analyses suggested increased reporting of panic attacks with semaglutide, but the association was substantially attenuated after adjustment for age, sex and indication.
- In analyses restricted to the period before July 2023, reporting was elevated in crude analyses, but the adjusted estimates were no longer statistically significant after adjustment for age, sex and indication. This attenuation is compatible with confounding, although a pharmacological effect cannot be excluded.
- Triangulation with randomized trials and active‐comparator observational studies did not support a consistent increase in panic attacks or related anxiety outcomes with semaglutide.
- This study shows how active‐comparator restricted disproportionality analysis, temporal assessment of reporting dynamics and evidence triangulation can improve the interpretation of post‐marketing safety signals in pharmacoepidemiology.

## Plain Language Summary

This study investigated whether the diabetes and weight‐loss drug semaglutide (commonly known as Ozempic or Wegovy) increases the risk of panic attacks. Researchers analyzed patient safety data from the FDA Adverse Event Reporting System (FAERS) spanning 2018 through 2024. While the raw numbers initially showed that panic attacks were reported more often with semaglutide than with other medications (like dulaglutide or empagliflozin), this connection weakened significantly once researchers adjusted for patient age, sex and the specific reason for taking the drug. Crucially, the surge in panic attack reports occurred only after mid‐2023, coinciding with heightened media attention and regulatory reviews regarding the mental health impacts of GLP‐1 drugs. This pattern is consistent with ‘notoriety bias’, meaning that greater public awareness may have led to more reports rather than the drug itself causing more panic attacks. However, this type of data can neither prove nor rule out a true effect.

Furthermore, a review of 12 studies, including pooled clinical trial data and real‐world observational studies, found no consistent evidence linking semaglutide to increased panic attacks or anxiety. Overall, the available evidence does not show a consistent link between semaglutide and panic attacks, although an association with panic attacks cannot be excluded.

## 1 Introduction

### 1.1 Background

Semaglutide is a long‐acting glucagon‐like peptide‐1 receptor agonist (GLP‐1 RA) approved for Type 2 diabetes and chronic weight management [1, 2]. Its rapid uptake reflects robust glycemic control, sustained weight reduction and cardiovascular benefit demonstrated in the SUSTAIN, STEP and SELECT trials, alongside increasing off‐label use and widespread public visibility [1, 2, 3, 4]. In pivotal randomized trials, psychiatric adverse events were rare and balanced between semaglutide and placebo arms; however, these trials were not designed or powered to detect rare, acute neuropsychiatric outcomes such as panic attacks [5, 6].

Post‐marketing pharmacovigilance analyses have reported signals of disproportionate reporting of acute psychiatric events, including panic attacks, with GLP‐1 RAs, particularly semaglutide [7, 8]. These observations emerged in parallel with heightened regulatory attention, including an Icelandic Medicines Agency alert and a subsequent European Medicines Agency Pharmacovigilance Risk Assessment Committee (PRAC) review initiated in July 2023, Report Number: EMA/PRAC/258271/2023, with a parallel evaluation by the US Food and Drug Administration [9, 10]. The FDA's preliminary evaluation in January 2024 did not identify evidence of a causal association with suicidality. Following a comprehensive review, the FDA requested removal of the suicidal behavior and ideation warning from affected weight‐management products in January 2026 [9]. The specific signal for acute panic attacks remains incompletely characterized in post‐marketing surveillance.

Spontaneous reporting data are difficult to interpret for this question. Crude disproportionality estimates are vulnerable to confounding by indication, because GLP‐1 RA users differ systematically from comparator populations in cardiometabolic risk, sex distribution and age structure [11, 12]. They are also vulnerable to notoriety bias, the rise in reporting that follows regulatory communications and media attention [13, 14, 15]. Most prior analyses have compared against heterogeneous all‐drug reference groups, which limits the interpretability of indication‐relevant comparisons [16].

For rare adverse drug effects, well‐conducted observational studies using active‐comparator, new‐user designs provide the most appropriate basis for risk quantification [12]. Randomized trials may be underpowered for rare acute neuropsychiatric outcomes, whereas spontaneous‐reporting systems lack exposure denominators and detailed clinical information. Disproportionality analysis therefore serves a narrower, hypothesis‐generating purpose and cannot quantify risk or establish causality [17]. Accordingly, our interpretation triangulates evidence across these sources while giving the greatest weight to studies whose designs and data are best suited to support causal inference.

GLP‐1 receptors are expressed in hypothalamic and limbic circuits involved in stress and affect regulation [18, 19, 20]. Preclinical work suggests that acute GLP‐1 receptor activation may engage hypothalamic–pituitary–adrenal and sympathetic pathways and induce anxiety‐like behaviour in animal models [21, 22, 23], whereas human data suggest neutral or potentially beneficial neuropsychiatric effects with chronic exposure [24, 25].

No study has yet evaluated panic attack as a discrete outcome using an active‐comparator restricted design with adjustment for indication and prespecified temporal restriction to address notoriety bias. We therefore conducted such an analysis of FAERS, complemented by a systematic review of randomized and observational evidence, using the semaglutide–panic attack pair as a case example of the difficulty of interpreting high‐profile pharmacovigilance signals in widely used medicines.

## 2 Methods

To address this objective, we conducted an active‐comparator restricted disproportionality analysis (ACR‐DA) using the FDA Adverse Event Reporting System (FAERS) and a systematic literature review.

### 2.1 Active‐Comparator Restricted Disproportionality Analysis

#### 2.1.1 Data Source

The ACR‐DA was reported in accordance with the Reporting of a Disproportionality Analysis for Drug Safety Signal Detection Using Individual Case Safety Reports in PharmacoVigilance (READUS‐PV) guidance (Tables S1 and S2) [17]. FAERS is a publicly available spontaneous reporting database maintained by the FDA. Reporting is mandatory for pharmaceutical manufacturers and voluntary for healthcare professionals and patients, with submissions made through standardized MedWatch forms or electronic reporting systems [26]. Quarterly data files were obtained from the publicly accessible FAERS dashboard for the period January 1, 2018, to December 31, 2024, corresponding to the period of increasing global uptake of semaglutide [27].

#### 2.1.2 Exposed and Active‐Comparator Groups

The exposed cohort comprised ICSRs in which semaglutide (marketed as Ozempic, Wegovy or Rybelsus) was identified as the primary suspect drug. Two active comparators were selected to reflect overlapping clinical indications and mitigate confounding by indication at the design stage. Dulaglutide is a within‐class GLP‐1 receptor agonist that shares semaglutide's mechanism of action and has overlapping use in Type 2 diabetes. This comparison was intended to provide class‐relevant context and help assess whether the reporting pattern was specific to semaglutide or shared within the class. Empagliflozin is a sodium‐glucose cotransporter‐2 inhibitor used in a broadly overlapping Type 2 diabetes population but without GLP‐1 receptor activity. This comparison was intended to provide context relative to a pharmacologically distinct diabetes treatment while improving comparability with respect to indication. Because the direction and magnitude of bias in a disproportionality analysis can depend on the comparator selected [12], both contrasts are reported. Orlistat, approved for weight management and linagliptin, a dipeptidyl peptidase‐4 inhibitor used for Type 2 diabetes, were also considered but were excluded because each had fewer than three panic‐attack reports, precluding sufficiently stable estimation. Labelled indications are presented in Table S3.

#### 2.1.3 Outcome Definition and Data Cleaning

The primary outcome was the MedDRA preferred term ‘panic attack’. Related MedDRA terms (e.g., ‘panic reaction’) were excluded to ensure outcome specificity and case definition consistency. Duplicate cases were identified and removed using the unique case identifiers provided by the OpenFDA platform [28]. Adverse events were analyzed at the individual report level. Only ICSRs in which the medication of interest was classified as the primary suspect were retained. Drug names were normalized using regular expressions and mapped against standardized vocabularies, including RxNorm and the Observational Health Data Sciences and Informatics framework, with RxNorm Concept Unique Identifiers, generic names and Anatomical Therapeutic Chemical codes added to enrich the dataset [29, 30]. Clinical characteristics extracted from each ICSR included age, sex, indication for use, time to panic attack, reporting country and serious outcomes.

#### 2.1.4 Statistical Analysis

Crude reporting odds ratios (RORs) with 95% confidence intervals were estimated using logistic regression. Bayesian disproportionality was assessed using the information component (IC) and its 95% lower bound (IC_025_) [31, 32]. A safety signal was defined when three criteria were jointly met: (i) the lower bound of the 95% CI for the ROR exceeded 1.0; (ii) IC_025_ exceeded 0; and (iii) at least three panic attack reports were available [33, 34].

Multivariable logistic regression was used to estimate adjusted reporting odds ratios (aRORs). Models included adjustment for covariates serving as proxies for confounding by indication, including sex (male as reference), age category (0–18, 19–44, 45–64 and ≥ 65 years; reference ≥ 65) and indication‐related variables (Type 2 diabetes and obesity/weight management) [35]. Because age and sex were unrecorded for a substantial proportion of reports, reports with unknown values were retained as separate categories in the multivariable models to preserve the full analytic sample. Indication was modelled using indicator variables for Type 2 diabetes and obesity or weight management; reports with neither indication recorded, including reports with another or an unrecorded indication, formed the reference category.

To mitigate confounding by indication, we conducted the primary analyses within a restricted active‐comparator framework, comparing semaglutide with dulaglutide (a within‐class GLP‐1 receptor agonist) and empagliflozin (a sodium‐glucose cotransporter‐2 inhibitor).

To account for potential notoriety bias following the July 2023 European Medicines Agency Pharmacovigilance Risk Assessment Committee investigation, we pre‐specified three complementary temporal strategies. First, pre‐warning analyses restricted to reports received before July 1, 2023, the prespecified analytic cut point marking the beginning of the month in which the review was initiated. Second, a drug‐by‐period comparison of 2018–2022 with 2023–2024, using whole calendar years because annual reporting proportions are the natural unit for that comparison. Third, we reported annual proportions of panic attack reports per 10 000 total reports for semaglutide and each comparator, stratified by those periods.

As a sensitivity analysis, we repeated the disproportionality analysis using all other primary‐suspect drugs reported in FAERS during 2018Q1–2024Q4 as the reference set. This analysis was intended to assess whether the temporal pattern observed in the active‐comparator analyses was robust to the use of a broader, heterogeneous reference set. We estimated crude and adjusted RORs for the full study period and separately before and on or after July 1, 2023. Adjusted models included age category, sex, Type 2 diabetes and obesity or weight‐management indication. We tested semaglutide‐by‐period interactions using both the July 1, 2023, cut point and calendar periods of 2018–2022 versus 2023–2024.

All analyses were performed in SAS version 9.4 (SAS Institute Inc., Cary, NC, USA), with visualization in R version 4.5.3.

### 2.2 Systematic Review

#### 2.2.1 Protocol Registration and Search Strategy

The systematic review was conducted in accordance with the Preferred Reporting Items for Systematic Reviews and Meta‐Analyses (PRISMA) 2020 statement (Tables S4 and S5) [36]. The protocol was prospectively registered in the International Prospective Register of Systematic Reviews (PROSPERO; CRD420251183110).

MEDLINE (Ovid), Embase (Ovid) and the Cochrane Central Register of Controlled Trials (CENTRAL) were searched from inception, with the searches completed between November 21 and November 24, 2025. The search combined controlled vocabulary and free‐text terms for semaglutide and GLP‐1 receptor agonists with terms for panic attacks, panic disorder and anxiety. We applied no study‐design or language filters in the database searches. As prespecified in the registered protocol, we excluded studies not published in English during screening. The complete search syntax, database coverage and execution dates and numbers of records retrieved are provided in Tables S6–S8.

#### 2.2.2 Inclusion and Exclusion Criteria

Studies were eligible if they enrolled human adults aged 18 years or older who were exposed to semaglutide by any route, formulation or indication and reported panic attacks or acute anxiety outcomes. Eligible designs included randomized controlled trials, observational cohort and cross‐sectional studies, pharmacovigilance analyses and case reports or case series. Studies evaluating GLP‐1 receptor agonists collectively were eligible only when semaglutide‐specific findings could be extracted. We excluded reviews, editorials, grey literature, in vitro and animal‐only studies, studies restricted to pregnant populations, ongoing trials without publicly available results and studies not published in English. Two reviewers independently screened titles, abstracts and full texts against the prespecified criteria, with disagreements resolved by consensus or third‐party adjudication.

#### 2.2.3 Outcomes of Interest

The primary outcome was panic attacks, defined as abrupt surges of intense fear peaking within minutes according to DSM‐5 criteria [37]. Because panic attacks are frequently captured under broader anxiety‐related terminology in clinical and pharmacovigilance datasets, studies reporting acute anxiety or anxiety‐related outcomes were also included.

#### 2.2.4 Study Selection and Data Extraction

Records were managed in Covidence (Veritas Health Innovation, Melbourne, Australia). After de‐duplication, two reviewers (B.M. and M.P.‐Z.) independently screened titles, abstracts and full texts against predefined eligibility criteria, with disagreements resolved by consensus or third‐party adjudication (F.T.M.). Data were extracted using a standardized form capturing study characteristics, population details, exposure and comparator definitions, outcome measures and effect estimates with corresponding 95% confidence intervals.

#### 2.2.5 Risk of Bias Assessment

Risk of bias and methodological quality were assessed independently by two reviewers (B.M. and M.A.O.), with disagreements resolved by consensus. The Cochrane Risk of Bias 2 tool was used for randomized trials [38]. ROBINS‐I was used to assess risk of bias in estimates of comparative intervention effects from non‐randomized comparative cohort studies [39]. For each study and outcome, we specified a target trial and the effect estimate of interest before making domain‐level risk‐of‐bias judgements. For single‐arm before‐after studies and cross‐sectional surveys that did not estimate comparative intervention effects within a target‐trial framework, methodological quality and design limitations were assessed using the modified Downs and Black checklist [40]. The Naranjo scale was used to assess causal attribution in case reports. For pharmacovigilance analyses, we considered the choice of reference group, covariate adjustment, duplicate‐report management, outcome definition and susceptibility to underreporting and stimulated reporting.

#### 2.2.6 Data Synthesis

Given heterogeneity in study designs, comparators and outcome definitions, findings were synthesized narratively by study type. Where sufficient data were available, effect estimates were recalculated and expressed as risk ratios with 95% confidence intervals. Analyses were performed in R (version 4.5.3; R Foundation for Statistical Computing, Vienna, Austria).

## 3 Results

### 3.1 ACR‐DA

#### 3.1.1 Study Population

After de‐duplication and restriction to primary‐suspect reports, 33 267 semaglutide, 54 282 dulaglutide and 25 380 empagliflozin individual case safety reports (ICSRs) were included from 2018Q1 to 2024Q4 (Table 1).

**TABLE 1:** Baseline demographic and clinical characteristics of reports associated with semaglutide, dulaglutide and empagliflozin from the FDA Adverse Event Reporting System (FAERS) database (2018Q1 to 2024Q4).

Characteristic	Semaglutide	Dulaglutide	Empagliflozin
Total reports	33 267	54 282	25 380
Panic attack reports, *n* (%)	87 (0.26)	28 (0.05)	13 (0.05)
Age (years)			
Median (IQR)	61.0 (51.0–70.0)	63.0 (55.0–71.0)	66.0 (57.0–74.0)
≤ 18, *n* (%)	17 (0.1)	10 (0.0)	1 (0.0)
19–44, *n* (%)	2565 (7.7)	1548 (2.9)	1019 (4.0)
45–64, *n* (%)	7246 (21.8)	8692 (16.0)	5323 (21.0)
≥ 65, *n* (%)	6837 (20.6)	8439 (15.5)	7665 (30.2)
Unknown, *n* (%)	16 602 (49.9)	35 593 (65.6)	11 372 (44.8)
Sex			
Male, *n* (%)	10 784 (32.4)	20 401 (37.6)	11 723 (46.2)
Female, *n* (%)	20 497 (61.6)	27 828 (51.3)	9998 (39.4)
Unknown, *n* (%)	1986 (6.0)	6053 (11.2)	3659 (14.4)
Indication of use			
Obesity/weight, *n* (%)	4099 (12.3)	232 (0.4)	17 (0.1)
Diabetes, *n* (%)	10 441 (31.4)	20 236 (37.3)	10 840 (42.7)
Serious outcomes			
Death, *n* (%)	397 (1.2)	773 (1.4)	1109 (4.4)
Disabling, *n* (%)	594 (1.8)	216 (0.4)	243 (1.0)
Hospitalization, *n* (%)	4710 (14.2)	3459 (6.4)	6263 (24.7)
Life‐threatening, *n* (%)	583 (1.8)	221 (0.4)	1603 (6.3)
Other/not serious, *n* (%)	26 983 (81.1)	49 613 (91.4)	16 162 (63.7)
Reporting country			
United States, *n* (%)	26 449 (79.5)	51 294 (94.5)	16 493 (65.0)
Great Britain, *n* (%)	803 (2.4)	214 (0.4)	1111 (4.4)
Germany, *n* (%)	165 (0.5)	230 (0.4)	1144 (4.5)
Canada, *n* (%)	604 (1.8)	41 (0.1)	761 (3.0)
Other, *n* (%)	3299 (9.9)	2272 (4.2)	4993 (19.7)
Unknown, *n* (%)	1947 (5.9)	231 (0.4)	878 (3.5)

*Note:* Total reports for each drug are indicated. Panic attack reports are shown as *n* (%) for each drug.

Abbreviation: IQR, interquartile range.

Panic attacks were reported in 87 semaglutide ICSRs (0.26%), 28 dulaglutide ICSRs (0.05%) and 13 empagliflozin ICSRs (0.05%). Baseline demographic, indication and reporting characteristics are summarized in Table 1.

#### 3.1.2 Signal Detection (2018–2024)

Compared with dulaglutide, semaglutide was associated with increased reporting of panic attacks in crude analyses (ROR, 5.08; 95% CI, 3.32–7.78; IC_025_, 0.57) (Table 2; Figure 1).

**TABLE 2:** Crude and adjusted reporting odds ratios for panic‐attack reports with semaglutide compared with two active comparators (dulaglutide and empagliflozin) and with all other primary‐suspect drugs in FAERS (full reference set), 2018Q1–2024Q4.

Drug of interest	Comparator	Crude IC (IC_025_)	Crude ROR (95% CI)	aROR Model 1 (95% CI)^a^	aROR Model 2 (95% CI)^b^
Semaglutide	Dulaglutide	0.99 (0.57)	5.08 (3.32–7.78)	2.82 (1.74–4.56)	1.38 (0.69–2.75)
Semaglutide	Empagliflozin	0.62 (0.03)	5.12 (2.86–9.17)	2.75 (1.41–5.36)	1.14 (0.48–2.68)
Semaglutide	All other primary‐suspect drugs	0.88 (0.58)	1.85 (1.50–2.29)	1.48 (1.18–1.87)	0.76 (0.48–1.18)

Abbreviations: aROR, adjusted reporting odds ratio; CI, confidence interval; IC, information component; ROR, reporting odds ratio.

A positive disproportionality signal was defined as a lower bound of the 95% confidence interval for the ROR > 1.0, an IC_025_ > 0 and at least three panic‐attack reports.

Full‐reference analyses compared semaglutide with all other primary‐suspect drugs reported in FAERS during 2018Q1–2024Q4. Active‐comparator and full‐reference estimates depend on different reference distributions and address complementary potential sources of bias. Estimates for reports received on or after July 1, 2023, are reported in the Section 3.

^a^ Model 1 (full study period): adjusted for age category, sex and indication (indicator variables for Type 2 diabetes and for obesity or weight management) across the entire 2018–2024 study period.

^b^ Model 2 (pre‐warning period): restricted to reports received before July 1, 2023, to reduce the potential influence of notoriety bias and adjusted for the same covariates as Model 1.

**FIGURE 1:**
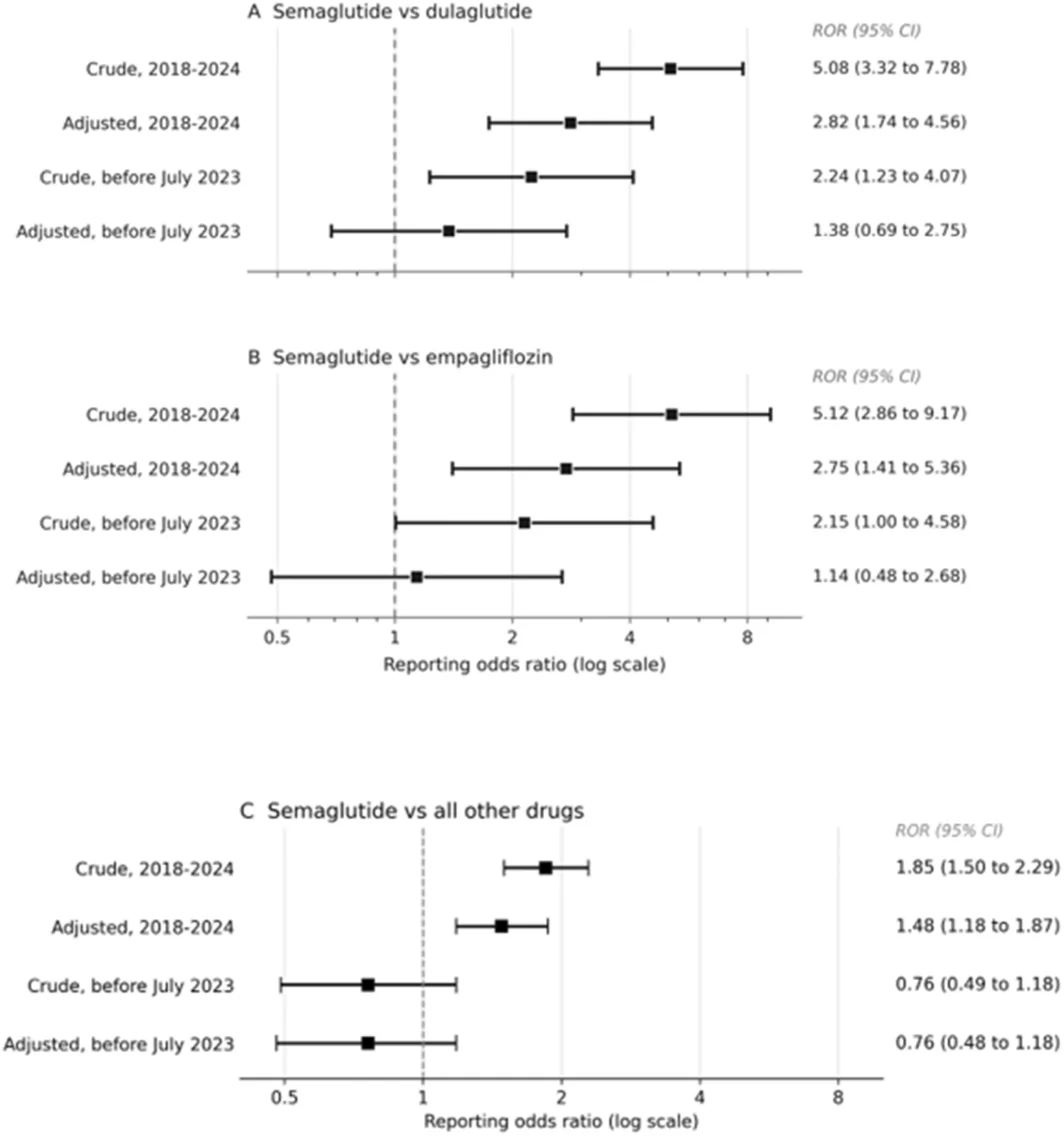
Crude and adjusted reporting odds ratios for panic attack reports with semaglutide compared with active comparators and with all other primary‐suspect drugs in FAERS (2018Q1 to 2024Q4). Panel A compares semaglutide with dulaglutide, Panel B with empagliflozin and Panel C with all other primary‐suspect drugs in FAERS (full reference set). Four estimates are shown for each comparison: crude and covariate‐adjusted reporting odds ratios across the full study period and crude and covariate‐adjusted reporting odds ratios restricted to reports received before July 1, 2023. Adjusted models include age category, sex and baseline indication (Type 2 diabetes; obesity/weight management). Squares represent ROR point estimates and horizontal bars indicate 95% confidence intervals. The vertical reference line is at ROR = 1.0. CI, confidence interval; FAERS, FDA Adverse Event Reporting System; ROR, reporting odds ratio.

Compared with empagliflozin, the crude ROR was 5.12 (95% CI 2.86–9.17; IC_025_ 0.03) (Table 2; Figure 1).

After adjustment for age, sex and indication, the adjusted ROR was 2.82 (95% CI, 1.74–4.56) versus dulaglutide and 2.75 (95% CI, 1.41–5.36) versus empagliflozin (Table 2; Figure 1).

#### 3.1.3 Temporal Analyses

##### 3.1.3.1 Pre‐Warning Analysis (Before July 1, 2023)

In analyses restricted to reports received before July 1, 2023, crude RORs for semaglutide were 2.24 (95% CI, 1.23–4.07) versus dulaglutide and 2.15 (95% CI, 1.00–4.58) versus empagliflozin. After adjustment for age, sex and indication, these estimates attenuated to 1.38 (95% CI, 0.69–2.75) and 1.14 (95% CI, 0.48–2.68), respectively and were no longer statistically significant (Table 2; Figure 1). Panic‐attack reports accounted for 0.108% of semaglutide reports received before July 1, 2023 and 0.262% of all semaglutide reports in the full study period, whereas the corresponding proportions were 0.048% and 0.052% for dulaglutide, 0.051% in both periods for empagliflozin and 0.142% and 0.140% for all other primary‐suspect drugs (Figure 2).

**FIGURE 2:**
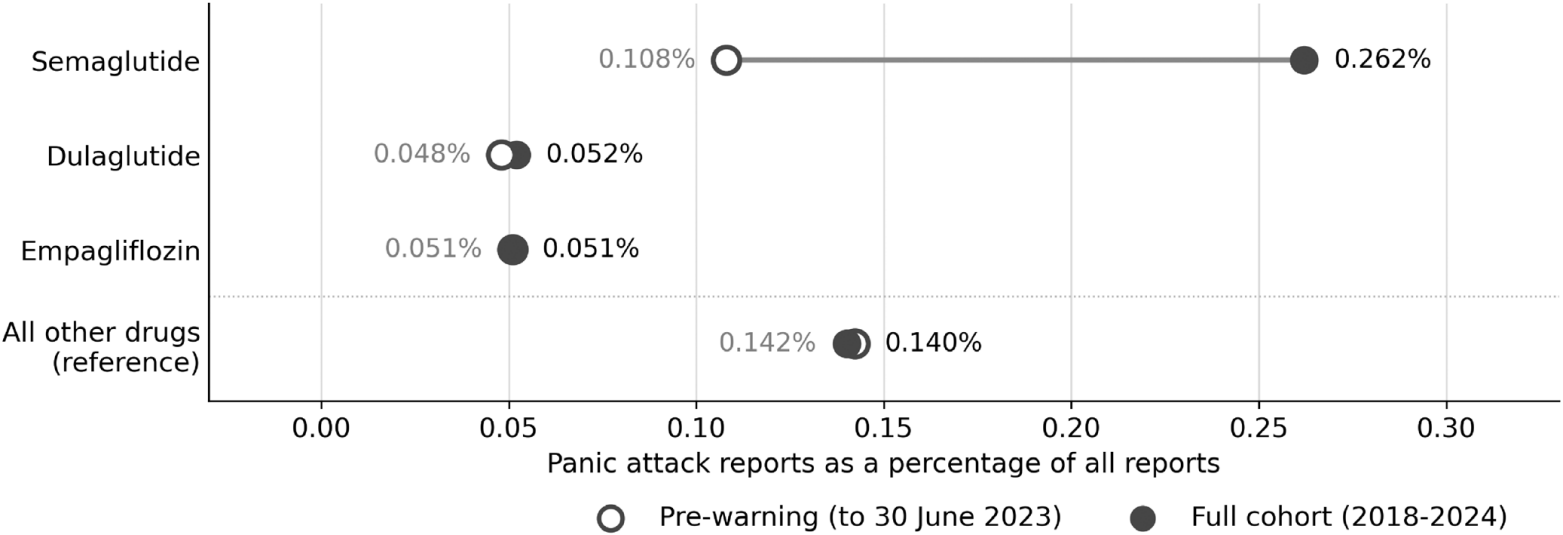
Crude proportion of panic attack reports among all adverse event reports in FAERS for semaglutide, dulaglutide, empagliflozin and all other primary‐suspect drugs in FAERS (full reference set) in the pre‐warning period (January 1, 2018 to June 30, 2023) and across the full cohort (January 1, 2018 to December 31, 2024). Semaglutide reporting proportions increased from 0.108% in the pre‐warning period to 0.262% in the full cohort, whereas proportions remained stable for dulaglutide (0.048% and 0.052%), empagliflozin (0.051% in both periods) and the full reference set (0.142% and 0.140%).

##### 3.1.3.2 Drug‐By‐Period Interaction Analysis

A significant drug‐by‐period interaction was observed in the comparison of 2018–2022 with 2023–2024 (*p* = 0.007) (Figure 3).

**FIGURE 3:**
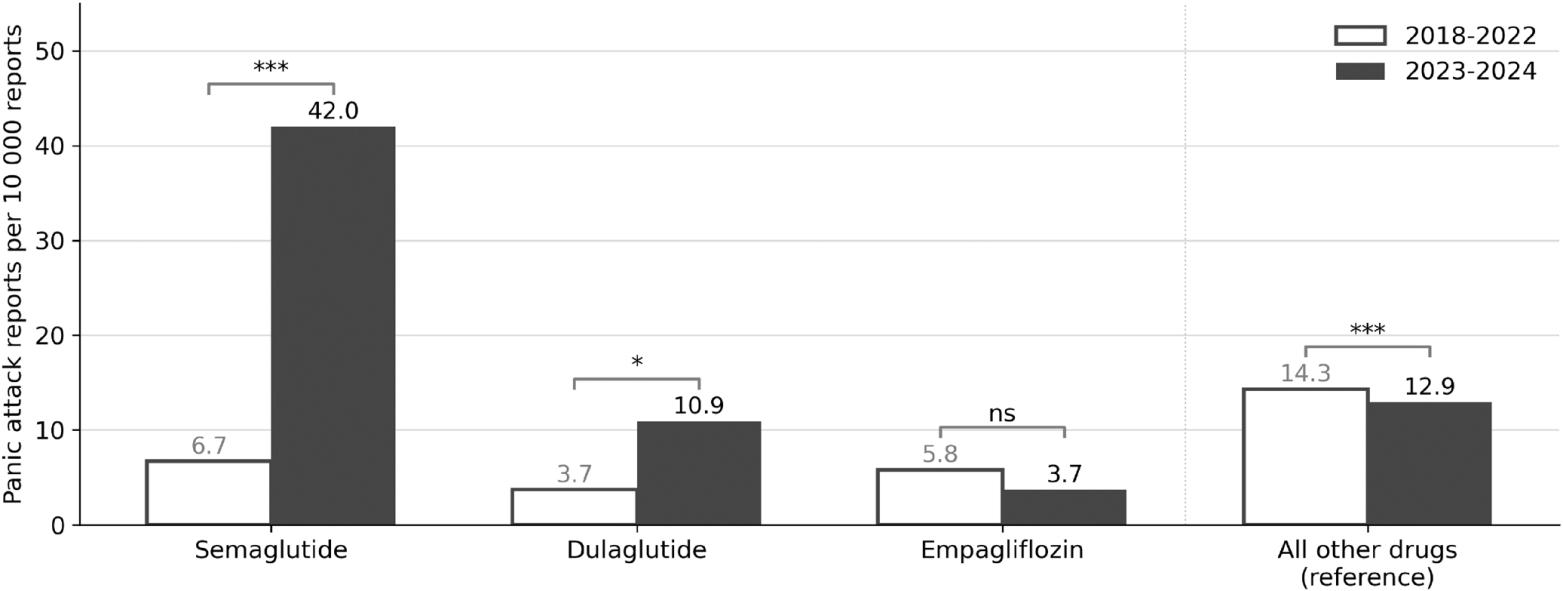
Panic attack reporting proportion per 10 000 FAERS reports for semaglutide, dulaglutide, empagliflozin and all other primary‐suspect drugs in FAERS (full reference set) across two periods, 2018–2022 and 2023–2024. Significance markers indicate the change in reporting odds between periods within each drug or the reference set, derived from a logistic regression model including a drug‐by‐period interaction term (*p < 0.05, ***p < 0.001, ns not significant). The drug‐by‐period interaction across semaglutide, dulaglutide and empagliflozin was statistically significant (*p* = 0.007). In the full‐reference analysis, the semaglutide‐by‐period interaction was also statistically significant (*p* < 0.001).

For semaglutide, the odds ratio for panic attack reporting in 2023–2024 versus 2018–2022 was 6.31 (95% CI, 3.26–12.20). For dulaglutide, the corresponding odds ratio was 2.95 (95% CI, 1.39–6.23). For empagliflozin, the odds ratio was 0.65 (95% CI, 0.18–2.35).

##### 3.1.3.3 Annual Reporting Patterns

Annual reporting proportions of panic attack ICSRs per 10 000 total reports for semaglutide were near zero in 2018.

Annual proportions for dulaglutide ranged from 0 to 11.8 per 10 000 across the study period and those for empagliflozin ranged from 0 to 5 per 10 000. Aggregated by calendar period, the proportion rose from 6.7 per 10 000 reports in 2018–2022 to 42.0 in 2023–2024 for semaglutide and from 3.7 to 10.9 for dulaglutide and fell from 5.8 to 3.7 for empagliflozin (Figure 3). In the full reference set of all other primary‐suspect drugs, the corresponding proportions were 14.3 and 12.9 per 10 000 reports (Figure 3).

#### 3.1.4 Full‐Reference Analysis

The full‐reference analysis included 33 267 semaglutide reports and 9 518 893 reports involving all other primary‐suspect drugs. For the full study period, the crude ROR for semaglutide was 1.85 (95% CI, 1.50–2.29; IC, 0.88; IC_025_, 0.58) and the adjusted ROR was 1.48 (95% CI, 1.18–1.87) (Table 2; Figure 1). Before July 1, 2023, the crude and adjusted RORs were 0.76 (95% CI, 0.49–1.18) and 0.76 (95% CI, 0.48–1.18), respectively (Table 2; Figure 1). On or after July 1, 2023, the corresponding estimates were 3.50 (95% CI, 2.74–4.46) and 2.15 (95% CI, 1.57–2.93), respectively. Semaglutide‐by‐period interactions were statistically significant using both the July 1, 2023, cut point and the comparison of 2018–2022 with 2023–2024 (both *p* < 0.001) (Figure 3).

### 3.2 Systematic Review

#### 3.2.1 Study Selection

Database searches identified 1002 records that met the eligible publication types (Embase *n* = 552; CENTRAL *n* = 342; MEDLINE *n* = 108). The CENTRAL search returned 496 records across all Cochrane Library segments, of which 342 were trial records; Cochrane Reviews, protocols and clinical answers were not eligible publication types and were not exported. After removal of 111 duplicates, 891 records underwent title and abstract screening, of which 845 were excluded.

Forty‐six full‐text articles were assessed for eligibility; 34 were excluded for incorrect outcome (*n* = 17), study design (*n* = 5), intervention (*n* = 1), language (*n* = 1) or ongoing studies without results (*n* = 10).

Twelve studies met eligibility criteria and were included in the qualitative synthesis (Figure 4; Tables S9–S12): one post hoc randomized trial analysis, six observational or cross‐sectional studies, four pharmacovigilance analyses and one case report [6, 8, 41, 42, 43, 44, 45, 46, 47, 48, 49, 50]. Risk of bias assessments are provided in Tables S13–S16.

**FIGURE 4:**
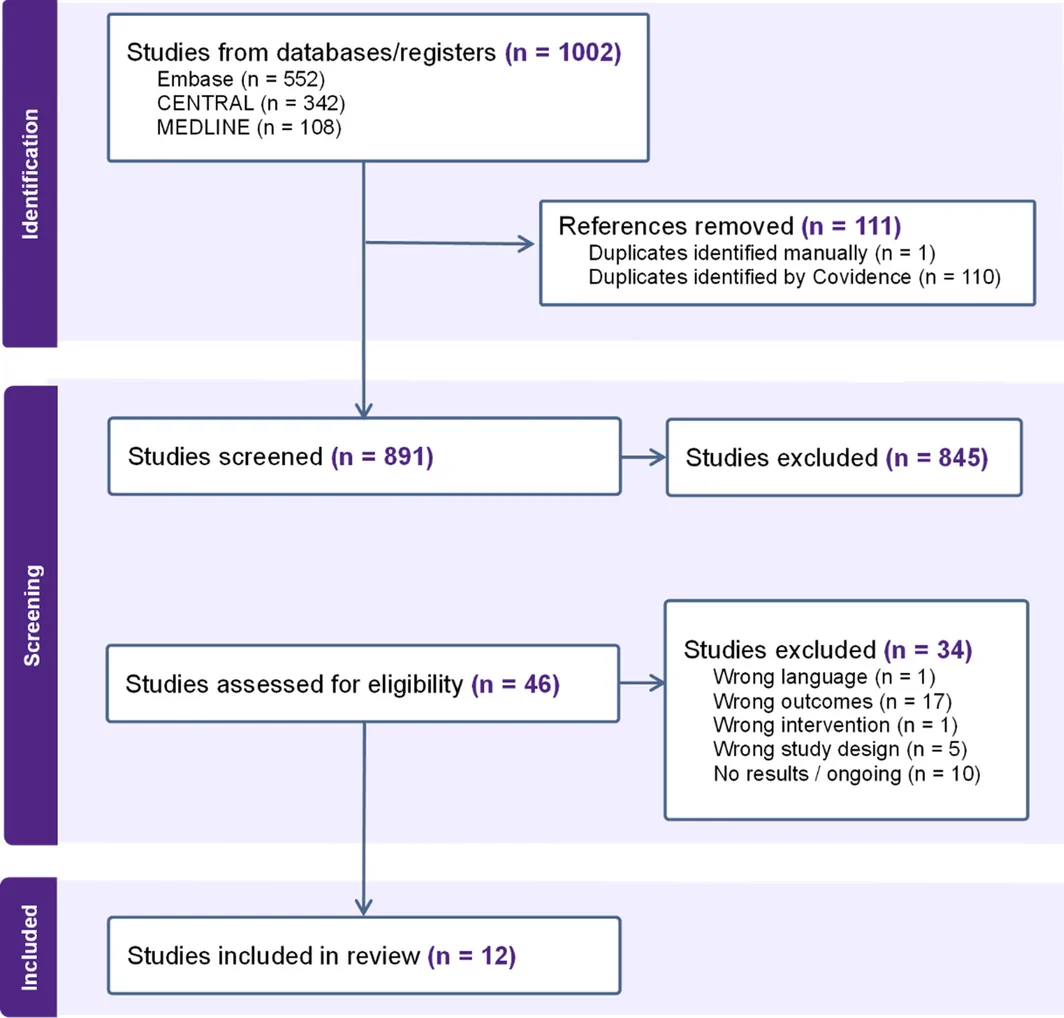
PRISMA 2020 flow diagram of study selection for the systematic review. Records were identified in MEDLINE, Embase and CENTRAL. Studies were eligible if the design was a randomized controlled trial, observational or cross‐sectional cohort study, pharmacovigilance disproportionality analysis or case report or case series; the intervention was semaglutide; and the outcome was panic attacks or acute anxiety.

The included studies were heterogeneous in design, population, comparator and outcome ascertainment. Only two of the 12 studies reported panic attacks as a discrete outcome and one of these included only five panic‐attack events. The remaining studies assessed broader outcomes, including anxiety diagnoses, anxiety‐related healthcare utilization or changes in anxiety symptom scores. These outcomes are not interchangeable with panic attacks. Given the clinical and methodological heterogeneity across studies, quantitative synthesis was not considered appropriate.

#### 3.2.2 Randomized Controlled Trials

Wadden et al. pooled data from the STEP 1, 2, 3 and 5 randomized, double‐blind, placebo‐controlled trials (*n* = 3377; semaglutide *n* = 2116; placebo *n* = 1261). Panic attacks occurred in 3 of 2116 participants receiving semaglutide and in 2 of 1261 receiving placebo (risk ratio, 0.89; 95% CI, 0.15–5.34). Anxiety disorders and symptoms (MedDRA high‐level group term) were reported in 75 of 2116 semaglutide‐treated participants and 50 of 1261 placebo‐treated participants (risk ratio, 0.89; 95% CI, 0.63–1.27). Risk of bias was assessed as low (RoB 2).

#### 3.2.3 Cohorts and Cross‐Sectional Studies

Six observational or cross‐sectional studies evaluated broader anxiety‐related outcomes—rather than panic attacks specifically—across administrative claims databases and survey cohorts.

In a propensity‐score matched cohort of 46 772 adults with Type 2 diabetes (TriNetX), De Giorgi et al. reported a hazard ratio of 0.92 (95% CI, 0.84–1.01) for incident anxiety disorders comparing semaglutide with sitagliptin (ROBINS‐I, moderate risk of bias).

In a difference‐in‐differences analysis of 774 968 adults with Type 2 diabetes (Komodo Healthcare Map), Do et al. reported a semaglutide‐specific incidence rate ratio of 0.81 (95% CI 0.76–0.87) for anxiety‐related office visits compared with dipeptidyl peptidase‐4 inhibitor use. Corresponding semaglutide‐specific estimates were 0.98 (95% CI 0.85–1.12) for emergency department visits, 1.26 (95% CI 0.90–1.77) for inpatient visits and 0.99 (95% CI 0.97–1.02) for outpatient hospital visits. This outcome reflected healthcare utilization for a broad group of anxiety diagnoses rather than panic attacks specifically (ROBINS‐I, moderate risk of bias).

In a cohort of 324 506 adults with obesity (TriNetX), Kornelius et al. reported a hazard ratio of 2.36 (95% CI 2.23–2.49) for incident anxiety in the semaglutide 2.4 mg subgroup compared with untreated obese controls (ROBINS‐I, serious risk of bias).

Two cross‐sectional surveys reported lower self‐reported anxiety among semaglutide users. A Polish survey (*n* = 1105) reported lower GAD‐7 scores among semaglutide users compared with non‐users and a Kuwaiti survey (*n* = 468) reported lower anxiety risk compared with liraglutide (risk ratio, 0.38; 95% CI, 0.22–0.67) and no difference versus tirzepatide (0.80; 95% CI, 0.41–1.55). Both surveys were susceptible to volunteer selection and self‐report biases; Witaszek et al. used the GAD‐7 scale, whereas Alali et al. used an unvalidated self‐report instrument (modified Downs and Black scores, 15/28 and 14/28, respectively). A German single‐arm before‐after study of 122 patients reported a reduction in HADS‐A scores over 1 year without a concurrent comparator (modified Downs and Black score, 14/28).

#### 3.2.4 Pharmacovigilance Studies

Four pharmacovigilance analyses evaluated spontaneous reporting databases (FAERS, VigiBase, DAEN).

In FAERS (2010–2024), Lu et al. reported crude reporting odds ratios (RORs) of 1.30 (95% CI 1.12–1.50) for anxiety in diabetes indications and 0.49 (95% CI 0.40–0.60) for weight‐control indications.

Chen et al. reported a ROR of 1.41 (95% CI, 1.23–1.63) for grouped psychiatric adverse events, with a median onset of 12 days (IQR, 4–45.5).

Katranski et al. reported a FAERS ROR of 1.46 (95% CI 1.16–1.82) for panic attacks and a DAEN ROR of 3.56 (95% CI 1.14–11.12).

In VigiBase, Nishida et al. reported an adjusted ROR of 1.26 (95% CI 1.18–1.35) for anxiety disorders, with a median onset of 7 days (IQR 1–33).

Of the four included pharmacovigilance analyses, three used unrestricted reference sets without adjustment. Nishida et al. used an active‐comparator design within antidiabetic and anti‐obesity medications with adjustment for age, sex and WHO region. None restricted the analysis temporally around the July 2023 regulatory communication and none reported panic attack as a discrete outcome adjusted for indication.

#### 3.2.5 Risk of Bias and Methodological Quality

The pooled randomized trial analysis was judged to have a low overall risk of bias using RoB 2, although the small number of panic‐attack events limited precision. Among the three non‐randomized comparative cohort studies assessed using ROBINS‐I, De Giorgi et al. and Do et al. were judged to have a moderate overall risk of bias for the relevant comparative effect estimates, whereas Kornelius et al. were judged to have a serious overall risk of bias, principally because of concerns regarding confounding, selection of participants into the study and measurement of outcomes. The single‐arm before‐after study and the two cross‐sectional surveys had important design and methodological limitations, as summarized using the modified Downs and Black checklist. The pharmacovigilance analyses were limited by spontaneous‐reporting biases and their inability to estimate incidence; three of the four used unrestricted reference groups without covariate adjustment. These methodological limitations reduce certainty in the observational evidence (Tables S13–S16; Figure S2).

#### 3.2.6 Case Report

A single case report described a male patient in his late 70s with Type 2 diabetes who developed acute anxiety, inner restlessness and distress 4 weeks after initiation of oral semaglutide 3 mg. Symptoms resolved within 9 days of discontinuation and initiation of sertraline and quetiapine. The Naranjo score was 7 (probable).

## 4 Discussion

### 4.1 Summary of Findings

We combined an ACR‐DA of FAERS with a systematic review of randomized and observational evidence to evaluate how confounding by indication and notoriety bias may shape the semaglutide–panic‐attack signal. Crude analyses suggested increased disproportional reporting. This association was substantially attenuated after adjustment for age, sex and indication and was no longer statistically significant in analyses restricted to the pre‐warning period before July 2023. Randomized trials and propensity‐score‐matched active‐comparator cohort studies did not suggest an increased risk of panic attacks or related anxiety outcomes with semaglutide, although event counts were low, trial estimates were imprecise and residual confounding may persist in observational cohorts. Taken together, our findings are more readily explained by confounding by indication and notoriety‐driven reporting than by a pharmacological effect, though an association with panic attacks cannot be excluded.

### 4.2 ACR‐DA

In the ACR‐DA, the initial disproportionality signal for semaglutide was reduced after adjustment for demographic and clinical characteristics and after restriction to the pre‐warning period. These findings are consistent with the possibility that differences in underlying patient characteristics, including age, sex distribution and indication (obesity versus diabetes), contributed to observed reporting patterns in spontaneous reporting data.

Several features of the post‐2023 reporting pattern suggest that it is more consistent with stimulated reporting than with a pharmacological effect. First, in the calendar‐period comparison, the proportion of semaglutide reports describing panic attacks increased from 6.7 per 10 000 reports in 2018–2022 to 42.0 per 10 000 reports in 2023–2024, corresponding to an OR of 6.31 (95% CI, 3.26–12.20). This increase coincided temporally with the 2023 EMA review and heightened public attention. Second, the increase was more pronounced for semaglutide than for the comparators: the corresponding OR was 2.95 (95% CI, 1.39–6.23) for dulaglutide and 0.65 (95% CI, 0.18–2.35) for empagliflozin, with evidence of a drug‐by‐period interaction (*p* = 0.007). Third, before July 2023, the adjusted RORs were 1.38 versus dulaglutide and 1.14 versus empagliflozin and were not statistically significant. Together, the timing and drug‐specificity of the increase are compatible with stimulated reporting [13, 14, 15]. However, FAERS lacks exposure denominators and these analyses cannot exclude contributions from changes in utilization, patient mix, event occurrence or other time‐varying factors. The temporal pattern should therefore be interpreted as suggestive rather than definitive evidence of notoriety bias.

In the sensitivity analysis using all other primary‐suspect drugs as the reference set, the temporal pattern was consistent with that observed in the active‐comparator analyses. No statistically significant disproportionality was observed before July 2023, whereas reporting was elevated thereafter, with statistically significant semaglutide‐by‐period interactions. This finding indicates that the observed post‐2023 temporal pattern was not dependent solely on the use of dulaglutide or empagliflozin as restricted active‐comparator reference groups.

The magnitude of the full‐period disproportionality estimate differed by reference set. The crude ROR for semaglutide was lower when compared with all other primary‐suspect drugs (ROR, 1.85) than when compared with dulaglutide (ROR, 5.08) or empagliflozin (ROR, 5.12). Panic attacks were reported in 14.0 per 10 000 reports in the full reference set, compared with approximately 5.2 per 10 000 dulaglutide reports and 5.1 per 10 000 empagliflozin reports. These differences demonstrate that the reporting profile of the selected comparator can materially influence the magnitude of a disproportionality estimate [12].

Thus, although the broader reference set attenuated the overall disproportionality estimate, the post‐2023 temporal pattern persisted and was compatible with, but not specific to, stimulated reporting. Changes in semaglutide utilization, patient characteristics, clinical recognition or coding of panic attacks, co‐reported conditions or medications, background event occurrence, media attention and other time‐varying factors may also have contributed. Conversely, because the full reference set includes drugs used across diverse indications and patient populations, it provides less control for confounding by indication and other between‐drug differences than an active‐comparator design. The active‐comparator and full‐reference analyses therefore address complementary concerns and should be interpreted jointly rather than as interchangeable estimates.

### 4.3 Systematic Review

The systematic review identified 12 studies with heterogeneous designs, populations and outcome definitions. The most consequential source of heterogeneity is the outcome itself. Only two studies reported panic attacks as a discrete outcome and one of these contributed five events in total. The remaining studies reported broader constructs, including anxiety diagnoses in claims or electronic health records, anxiety‐related healthcare utilization and self‐reported symptom scores, which are not interchangeable with panic attacks. Randomized trials from the STEP program showed no imbalance in either outcome but were not powered for rare events. Active‐comparator cohort studies generally reported null or inverse associations, whereas the study using an untreated comparator reported an elevated hazard ratio. Under ROBINS‐I, the latter study was judged to have serious risk of bias, particularly because of concerns about confounding, selection of participants into the study and outcome measurement. Compared with an active‐comparator design, an untreated‐comparator design may be more susceptible to residual confounding when treatment initiation is associated with baseline clinical characteristics, healthcare‐seeking behaviour or other factors related to anxiety risk. In addition, anxiety ascertainment based on diagnostic codes requires healthcare contact, which may differ between treated and untreated individuals. Consequently, this design was less able to distinguish a potential drug effect from differences in baseline risk, healthcare utilization or outcome ascertainment.

Pharmacovigilance studies using spontaneous reporting databases reported variable disproportionality estimates; however, only one used an active‐comparator design and none adjusted for indication or accounted for temporal reporting effects. Cross‐sectional survey data were subject to selection and reporting bias and uncontrolled cohort data did not permit causal inference. Substantial heterogeneity in outcome definitions limited comparability across studies and precluded quantitative synthesis. Overall, we interpreted these heterogeneous findings within a triangulation framework, evaluating the coherence of estimates across trials, cohort studies and pharmacovigilance analyses with FAERS patterns, while accounting for confounding by indication and notoriety bias. Interpretation prioritized evidence from randomized trials and active‐comparator cohort studies over pharmacovigilance estimates.

### 4.4 Comparison With Prior Studies

Our findings are broadly consistent with recent large observational studies using active‐comparator and longitudinal designs, which have not demonstrated an association between GLP‐1 receptor agonist use and serious psychiatric outcomes [51, 52]. In contrast, earlier spontaneous reporting analyses suggested potential signals; however, these studies generally relied on broad or ‘all‐drug’ comparators and did not apply active‐comparator restricted or temporally stratified designs, making them susceptible to confounding by indication and notoriety bias [8, 47, 48]. Differences in study design, comparator selection and temporal context likely account for discrepancies across studies.

### 4.5 Biological Considerations

GLP‐1 receptors are expressed in central nervous system regions involved in autonomic and affective regulation, including hypothalamic and limbic structures. Preclinical studies have shown that acute GLP‐1 receptor activation may engage stress‐related neuroendocrine pathways and induce anxiety‐like behavior in animal models [18, 19, 20]. However, clinical trial and observational data do not suggest consistent increases in anxiety‐related outcomes with therapeutic GLP‐1 receptor agonist use [24, 25]. The clinical relevance of mechanistic findings remains uncertain, particularly given differences between experimental exposure conditions and chronic clinical use.

### 4.6 Strengths and Limitations

This study has several strengths, including the use of an active‐comparator restricted design, prespecified temporal analyses to evaluate reporting dynamics and triangulation with a systematic review of randomized and observational evidence. The incorporation of temporal sensitivity analyses strengthens the interpretation of potential reporting bias in pharmacovigilance data. Importantly, we did not assume a single definitive reference standard for semaglutide‐associated panic attacks. Instead, we interpreted the FAERS signal alongside randomized trials and observational studies, placing greater emphasis on evidence from stronger causal inference designs, while recognizing that each data source addresses a different estimand.

Disproportionality analysis of spontaneous reports is a hypothesis‐generating method [17]. It compares the reporting of a particular event for a drug of interest with reporting for a specified comparator drug or group of drugs or with all other drugs in the database. It cannot estimate the incidence of panic attacks among people taking semaglutide because the number of exposed individuals is unknown and no population denominator is available. It also cannot establish that semaglutide caused any reported event and a reporting odds ratio should not be interpreted as a relative risk.

Spontaneous reporting systems are subject to underreporting and incomplete clinical information. Although underreporting does not necessarily bias a reporting odds ratio, bias may arise if panic attacks are preferentially reported for one drug relative to the comparator or if reporting patterns change over time, such as following regulatory communications or heightened public attention [29, 53].

Panic‐attack reports in FAERS are not routinely clinically verified and MedDRA preferred terms may not correspond to episodes meeting DSM‐5 criteria. Publicity surrounding a possible psychiatric safety concern could also influence how symptoms are recognized, described or coded, potentially introducing differential outcome misclassification. However, the direction and magnitude of any such misclassification cannot be determined from FAERS.

Missing data warrant particular emphasis. Age was unrecorded in approximately half of semaglutide reports and two‐thirds of dulaglutide reports; sex was missing in a smaller but differing proportion across drug groups and indication was recorded in fewer than half of reports in each group. The extent of missingness therefore differed between semaglutide and the comparators. Retaining unknown age and sex values as separate categories preserved the full sample but does not eliminate bias if covariate recording was related to the drug, the reported event, reporter characteristics or reporting route. Moreover, the limited recording of indication constrained our ability to address confounding by indication. Consequently, adjustment for age, sex and indication may have been incomplete and the adjusted reporting odds ratios should be interpreted cautiously.

Neither comparator is approved for weight management. Orlistat, the candidate comparator approved for weight management, was considered but could not be used because fewer than three panic‐attack reports were available. Obesity or weight management was recorded as the indication in 12.3% of semaglutide reports but in only 0.4% of dulaglutide and 0.1% of empagliflozin reports (Table 1). Adjustment for this indication therefore relies on very few comparator reports and residual confounding by indication among patients treated for weight management cannot be excluded.

The evidence identified in the systematic review provides limited certainty regarding panic attacks specifically. Only two studies evaluated panic attacks as a discrete outcome: a pooled trial analysis with five events in total and a pharmacovigilance analysis, which cannot estimate risk. Most observational studies assessed broader anxiety diagnoses, anxiety‐related healthcare utilization or symptom scales, which may not capture panic attacks and cannot be assumed to represent the same clinical outcome. The included studies also differed substantially in design, study population, comparator, follow‐up, outcome definition and risk of bias. Accordingly, the lack of a consistent increase in risk across this broader evidence base should be interpreted cautiously and does not exclude an association with panic attacks specifically.

Residual confounding by disease severity, psychiatric history and healthcare utilization may persist despite comparator restriction and covariate adjustment [29, 54, 55].

## 5 Conclusions

In this pharmacovigilance analysis, semaglutide was associated with increased reporting of panic attacks in crude FAERS analyses; however, this association was attenuated after adjustment for demographic and indication‐related factors and the adjusted estimates were not statistically significant in analyses restricted to reports received before July 1, 2023. Evidence from randomized trials and active‐comparator observational cohort studies did not suggest an increased risk of panic attacks or related anxiety outcomes with semaglutide.

Taken together, these findings are more consistent with confounding by indication and notoriety‐driven reporting within spontaneous reporting data than with a robust pharmacological signal. Nevertheless, given the inherent limitations of spontaneous reporting systems and observational evidence, residual uncertainty remains and further controlled pharmacoepidemiological studies are warranted.

This case study illustrates how active‐comparator disproportionality analysis, temporal assessment of reporting dynamics and evidence triangulation can improve the interpretation of post‐marketing safety signals for widely used medications.

## Author Contributions

**Ben Melamed:** conceptualization, formal analysis, investigation, methodology, visualization, writing original draft, writing review and editing. **Mahak Parsi‐Zahabi:** investigation, data curation. **Mohammad Ali Omrani:** methodology, investigation, data curation, writing review and editing. **Bala Swetha Baskaran:** investigation, data curation. **Lujain Ez Eddin:** investigation, data curation. **Nazgol Kermanshahi:** investigation, data curation. **Niaz Chalabianloo:** data curation, software. **Flory Tsobo Muanda:** conceptualization, supervision, writing review and editing, project administration.

## Funding

The authors have nothing to report.

## Conflicts of Interest

The authors declare no conflicts of interest.

## Supporting information


**Table S1:** READUS‐PV (Reporting of a Disproportionality Analysis for Drug Safety Signal Detection Using Individual Case Safety Reports in PharmacoVigilance) checklist for abstracts. Page numbers refer to the revised manuscript (clean version).
**Table S2:** READUS‐PV checklist (main). Page numbers refer to the revised manuscript (clean version).
**Table S3:** Comparison of labelled indications for the study drugs evaluated in the active‐comparator restricted disproportionality analysis.
**Table S4:** Preferred Reporting Items for Systematic Reviews and Meta‐Analyses (PRISMA) 2020 for Abstracts Checklist.
**Table S5:** Preferred Reporting Items for Systematic Reviews and Meta‐Analyses (PRISMA) 2020 Checklist. Page numbers refer to the revised manuscript (clean version).
**Table S6:** Full search strategy for Ovid MEDLINE(R) ALL (1946 to 20 November 2025).
**Table S7:** Full search strategy for Embase Classic and Embase (1947 to 19 November 2025).
**Table S8:** Full search strategy for the Cochrane Central Register of Controlled Trials (CENTRAL) (inception to 24 November 2025).
**Table S9:** Summary characteristics of clinical trials evaluating semaglutide and panic attacks.
**Table S10:** Summary characteristics of observational and cross‐sectional studies evaluating semaglutide and acute anxiety.
**Table S11:** Summary characteristics of retrospective pharmacovigilance disproportionality analyses evaluating semaglutide and psychiatric outcomes.
**Table S12:** Summary characteristics of clinical case reports evaluating semaglutide and acute psychiatric events.
**Table S13:** Risk of bias assessment of included randomized clinical trials using the Cochrane Risk of Bias 2 (RoB 2) tool.
**Table S14:** Risk of bias assessment of included non‐randomized comparative cohort studies using the Risk Of Bias In Non‐randomized Studies of Interventions (ROBINS‐I) tool.
**Table S15:** Methodological quality assessment of the included single‐arm before‐after study and cross‐sectional surveys using the modified Downs and Black checklist.
**Table S16:** Causality assessment of included case reports using the Naranjo Adverse Drug Reaction Probability Scale.
**Figure S1:** Flow diagram of FAERS individual case safety reports included in the active‐comparator restricted disproportionality analysis. ICSRs were identified for semaglutide, dulaglutide or empagliflozin from 2018Q1 to 2024Q4, de‐duplicated using the OpenFDA case identifier, restricted to reports in which the medication of interest was the primary suspect drug and cleaned via regular‐expression normalization mapped to RxNorm and the Observational Health Data Sciences and Informatics framework. Orlistat and linagliptin were considered as additional comparators but excluded because of insufficient panic attack reports (fewer than three panic attack reports each). Final cohort sizes and panic attack counts are shown.
**Figure S2:** Traffic‐light summary of ROBINS‐I risk of bias judgements for the three included non‐randomized comparative cohort studies. Domains are D1, bias due to confounding; D2, bias in selection of participants into the study; D3, bias in classification of interventions; D4, bias due to deviations from intended interventions; D5, bias due to missing data; D6, bias in measurement of outcomes; D7, bias in selection of the reported result. A target trial was specified for each study before assessment. Assessments were made independently by two reviewers (B.M. and M.A.O.), with disagreements resolved by consensus. Domain‐level judgements are given in Table S14. The single‐arm before‐after study and the two cross‐sectional surveys were not assessed with ROBINS‐I because they did not estimate comparative intervention effects within a target‐trial framework; these are presented in Table S15.

## Data Availability

The data that support the findings of this study are publicly available at https://open.fda.gov.
