# Supplementary material for: Semaglutide and Panic Attacks: Active‐Comparator Disproportionality Analysis, Temporal Assessment of Notoriety Bias and Evidence Triangulation in Post‐Marketing Pharmacovigilance
Source: Pharmacoepidemiol Drug Saf. 2026 Oct 27;35(11):e70517. doi: 10.1002/pds.70517 (PMC13713200; doi:10.1002/pds.70517)
Supplement: Supplementary file 1 [file PDS-35-e70517-s001.docx]

**Supplementary Material for:**

**Semaglutide and panic attacks: active-comparator disproportionality analysis, temporal assessment of notoriety bias, and evidence triangulation in post-marketing pharmacovigilance**

Subtitle: Semaglutide and Panic Attacks

Authors: Ben Melamed^1^, Mahak Parsi-Zahabi^1^, Mohammad Ali Omrani^1^, Bala Swetha Baskaran^1^, Lujain Ez Eddin^1^, Nazgol Kermanshahi^1^, Niaz Chalabianloo^1^, and Flory Tsobo Muanda^1,2,3,4*^

^1^Department of Physiology and Pharmacology, Western University, London, Ontario, Canada;

^2^ICES Western, Kidney, Dialysis & Transplantation Research Program, London, Ontario, Canada; ^3^Department of Epidemiology and Biostatistics, Western University, London, Ontario, Canada;

^4^London Health Sciences Centre, Lawson Health Research Institute, London, Ontario, Canada

***Corresponding author.** E-mail: [fmuandat@uwo.ca](mailto:fmuandat@uwo.ca)

**Contents**

**Table S1.** READUS-PV checklist for abstracts (Reporting of a Disproportionality Analysis for Drug Safety Signal Detection Using Individual Case Safety Reports in PharmacoVigilance)

**Table S2.** READUS-PV checklist (main)

**Table S3.** Comparison of labelled indications for the study drugs (semaglutide, dulaglutide, empagliflozin, orlistat, linagliptin)

**Table S4.** PRISMA 2020 for Abstracts Checklist

**Table S5.** PRISMA 2020 Checklist

**Table S6.** Full search strategy for Ovid MEDLINE(R) ALL (1946 to 20 November 2025)

**Table S7.** Full search strategy for Embase Classic and Embase (1947 to 19 November 2025)

**Table S8.** Full search strategy for the Cochrane Central Register of Controlled Trials (CENTRAL) (inception to 24 November 2025)

**Table S9.** Summary characteristics of clinical trials evaluating semaglutide and panic attacks

**Table S10.** Summary characteristics of observational and cross-sectional studies evaluating semaglutide and acute anxiety

**Table S11.** Summary characteristics of retrospective pharmacovigilance disproportionality analyses evaluating semaglutide and psychiatric outcomes

**Table S12.** Summary characteristics of clinical case reports evaluating semaglutide and acute psychiatric events

**Table S13.** Risk of bias assessment of included randomized clinical trials using the Cochrane Risk of Bias 2 (RoB 2) tool

**Table S14. Risk of bias assessment of included non-randomised comparative cohort studies using the Risk Of Bias In Non-randomised Studies of Interventions (ROBINS-I) tool**

**Table S15. Methodological quality assessment of the included single-arm before-after study and cross-sectional surveys using the modified Downs and Black checklist**

**Table S16. Causality assessment of included case reports using the Naranjo Adverse Drug Reaction Probability Scale**

**Figure S1. Flow diagram of FAERS individual case safety reports included in the disproportionality analysis**

**Figure S2. Traffic-light summary of ROBINS-I risk of bias judgements for the three included non-randomised comparative cohort studies**

**References Supporting references for the studies described in Tables S9 to S16**

**Table S1.** READUS-PV (Reporting of a Disproportionality Analysis for Drug Safety Signal Detection Using Individual Case Safety Reports in PharmacoVigilance) checklist for abstracts. Page numbers refer to the revised manuscript (clean version).

| **Section and topic** | **Item #** | **Checklist item** | **Location** |
| --- | --- | --- | --- |
| Background | 1a | State the aim/rationale for performing the study. | Page 2 |
|  | 1b | Specify the adverse event(s) and/or the drug(s) under study, when applicable. | Page 2 |
|  | 1c | Specify the specific population or setting, when applicable. | Page 2 |
| Methods | 2a | Identify the study as a disproportionality analysis and specify the type of data used. | Page 2 |
|  | 2b | Specify the name of the database(s) used and the type of access. | Page 2 |
|  | 2c | Specify the timeframe and geographical region, when applicable. | Page 2 |
|  | 2d | Specify the disproportionality measure(s) used and their statistical significance threshold(s). | Page 2 |
|  | 2e | Specify if a case-by-case analysis is performed. | Page 2 |
| Results | 3 | Report main findings including their precision (e.g., 95% confidence intervals), together with a short summary of the case-by-case analysis. | Pages 2-3 |
| Conclusion | 4a | Clearly report key conclusions. | Page 3 |
|  | 4b | Acknowledge that the disproportionality analysis is a hypothesis generating or refinement approach. | Page 3 |
|  | 4c | State the implications and clinical relevance of the findings. | Page 3 |

*From: Fusaroli M, Salvo F, Begaud B, et al. The reporting of a disproportionality analysis for drug safety signal detection using individual case safety reports in PharmacoVigilance (READUS-PV): explanation and elaboration. Drug Saf 2024; 47: 585-599.*

**Table S2.** READUS-PV checklist (main). Page numbers refer to the revised manuscript (clean version).

| **Section and topic** | **Item #** | **Checklist item** | **Location** |
| --- | --- | --- | --- |
| **Title** |  |  |  |
|  | 1a | If disproportionality analyses are a prominent component of the published study, the study should be identified as a disproportionality analysis. The type of data and name of the database(s) should be specified. | Page 1 |
|  | 1b | Report the name of adverse event(s) and/or drug(s) under study, when applicable. | Page 1 |
| **Introduction** |  |  |  |
| Background | 2a | Describe the drug(s) and its utilization, the nature of the adverse event(s) under study and its frequency, and the existing knowledge on the drug-event combination. | Page 6 |
|  | 2b | Specify the rationale for performing the analysis, e.g., as part of routine pharmacovigilance, to investigate an overall safety profile, or to assess a pre-specified hypothesis. | Page 6 |
|  | 2c | Explain why ICSR databases and disproportionality analysis are suitable to fill the knowledge gap. | Pages 6-7 |
| Objectives | 3 | State specific objectives, identifying the adverse event(s), the drug(s), and the reference group, including any pre-specified hypothesis, if applicable. | Page 7 |
| **Methods** |  |  |  |
| Study design | 4a | Identify the study (i.e., disproportionality analysis) and the type of data used (e.g., individual case safety reports). | Page 8 |
|  | 4b | Provide an outline of the entire study design, including primary and sensitivity analyses performed, and other designs such as case-by-case analysis or literature review. | Pages 8-11 |
| Data description, access, pre-processing | 5a | Specify the name of the database(s), the database custodian, and the coverage. Specify the type/number of drugs included within the database and the thesaurus, taxonomies, or ontologies used for coding drugs and events. | Page 8 |
|  | 5b | Specify the extraction dates and describe and justify all choices used for data pre-processing, including any data transformation or exclusion, if appropriate. | Page 8 |
| Variables definition | 6a | Describe the study population, including any restriction. | Pages 8-9 |
|  | 6b | Describe the nature and the meaning of key variables assessed in the work. | Page 9 |
|  | 6c | Specify and justify any grouping of drugs or events. For drugs, specify and justify whether active ingredients/trade names/salts were considered and/or the selected role. | Pages 8-9 |
|  | 6d | Describe any additional data source used, the type of data, and how they interact with ICSRs. | Not applicable |
| Statistical methods | 7a | Present any descriptive analysis performed, specifying variables investigated, statistical tests, and significance thresholds. | Page 10 |
|  | 7b | Describe the measure(s) selected for the disproportionality analysis including any threshold used to identify signals of disproportionate reporting. Explain the reason for this choice if applicable. | Page 10 |
|  | 7c | Clearly describe any sensitivity analysis and any tool to control confounding, including any restriction, subgroup, stratification, adjustment, or interaction. | Pages 10-11 |
|  | 7d | Specify the variables and methods used for the case-by-case analysis, including any algorithm or criteria used to assess causality, if performed. | Not applicable |
|  | 7e | Specify any statistical methods used for other data sources. | Page 10 |
| **Results** |  |  |  |
| Participants | 8a | Specify the number of individual case safety reports included at each stage, including reasons for exclusion. | Page 15; Figure S1 |
|  | 8b | Provide key demographic and clinical characteristics of cases, if possible comparing cases with any appropriate reference group. | Page 15; Table 1 |
| Disproportionality analysis | 9 | Present all results including confidence intervals. Present also results of sensitivity analyses, if performed. | Pages 15-17; Table 2; Figures 1-3 |
| Case-by-case analysis | 10 | Present the case-by-case analysis of key variables. Present the causality assessment, if applicable. | Not applicable |
| **Discussion** |  |  |  |
| Key results | 11 | Discuss key results with reference to study objectives and contextualize them within the current literature. | Pages 22-24 |
| External validity | 12a | Discuss the external validity of the results to the general population. | Page 25 |
|  | 12b | Discuss the potential relevance of results in clinical practice. | Page 29 |
|  | 12c | Propose further study designs if applicable. | Page 29 |
| Limitations | 13 | Present general limitations, making clear that disproportionality analysis alone cannot prove causation or measure incidence, and specific limitations, including confounding and reporting bias and efforts to mitigate them. | Pages 26-28 |
| **Declarations** |  |  |  |
|  | 14a | Provide the source of funding/sponsorship and the role of the funders/sponsors for the present study and for any original study on which the present article is based. | Page 29 |
|  | 14b | Clearly identify potential commercial and intellectual conflicts of interest. | Page 29 |
|  | 14c | Declare any institutional approval needed or granted in the investigation. | Not applicable |
|  | 14d | Include a statement on data availability, code availability, and protocol registration. | Page 30 |

*From: Fusaroli M, Salvo F, Begaud B, et al. The reporting of a disproportionality analysis for drug safety signal detection using individual case safety reports in PharmacoVigilance (READUS-PV): explanation and elaboration. Drug Saf 2024; 47: 585-599.*

**Table S3.** Comparison of labelled indications for the study drugs evaluated in the active-comparator restricted disproportionality analysis.

| **Drug** | **Class** | **Labelled indications** |
| --- | --- | --- |
| Semaglutide | GLP-1 receptor agonist | Type 2 diabetes mellitus (Ozempic 0.5 to 2.0 mg subcutaneous weekly; Rybelsus 3 to 14 mg oral daily); chronic weight management in adults with body mass index (BMI) >=30 kg/m^2^, or >=27 kg/m^2^ with at least one weight-related comorbidity (Wegovy 2.4 mg subcutaneous weekly); reduction of major adverse cardiovascular events in adults with established cardiovascular disease and overweight or obesity (Wegovy 2.4 mg). |
| Dulaglutide | GLP-1 receptor agonist | Type 2 diabetes mellitus (Trulicity 0.75 to 4.5 mg subcutaneous weekly); reduction of major adverse cardiovascular events in adults with type 2 diabetes who have established cardiovascular disease or multiple cardiovascular risk factors. |
| Empagliflozin | Sodium-glucose cotransporter-2 (SGLT2) inhibitor | Type 2 diabetes mellitus (Jardiance 10 to 25 mg oral daily); reduction of cardiovascular death in adults with type 2 diabetes and established cardiovascular disease; reduction of cardiovascular death and hospitalization for heart failure in adults with heart failure (across the ejection fraction spectrum); chronic kidney disease. |
| Orlistat | Pancreatic and gastric lipase inhibitor | Chronic weight management (Xenical 120 mg three times daily by prescription; Alli 60 mg three times daily over the counter) in adults with BMI >=30 kg/m^2^, or >=27 kg/m^2^ with at least one weight-related comorbidity, in conjunction with a reduced-calorie diet. |
| Linagliptin | Dipeptidyl peptidase-4 (DPP-4) inhibitor | Type 2 diabetes mellitus (Tradjenta 5 mg oral daily) as an adjunct to diet and exercise to improve glycaemic control. |

Reference: *UpToDate Lexidrug. Wolters Kluwer; 2026. Accessed February 2026.* Dulaglutide and empagliflozin were selected as active comparators because they share clinical indications with semaglutide. Orlistat and linagliptin were initially considered as additional active comparators but were excluded owing to insufficient panic attack reports (fewer than three panic attack reports each).

**Table S4.** Preferred Reporting Items for Systematic Reviews and Meta-Analyses (PRISMA) 2020 for Abstracts Checklist.

| **Section and Topic** | **Item #** | **Checklist item** | **Reported (Yes/No)** |
| --- | --- | --- | --- |
| **TITLE** |  |  |  |
| Title | 1 | Identify the report as a systematic review. | Yes |
| **BACKGROUND** |  |  |  |
| Objectives | 2 | Provide an explicit statement of the main objective(s) or question(s) the review addresses. | Yes |
| **METHODS** |  |  |  |
| Eligibility criteria | 3 | Specify the inclusion and exclusion criteria for the review. | Yes |
| Information sources | 4 | Specify the information sources (e.g. databases, registers) used to identify studies and the date when each was last searched. | Yes |
| Risk of bias | 5 | Specify the methods used to assess risk of bias in the included studies. | Yes |
| Synthesis of results | 6 | Specify the methods used to present and synthesise results. | Yes |
| **RESULTS** |  |  |  |
| Included studies | 7 | Give the total number of included studies and participants and summarise relevant characteristics of studies. | Yes |
| Synthesis of results | 8 | Present results for main outcomes, preferably indicating the number of included studies and participants for each. If meta-analysis was done, report the summary estimate and confidence/credible interval. If comparing groups, indicate the direction of the effect (i.e. which group is favoured). | Yes |
| **DISCUSSION** |  |  |  |
| Limitations of evidence | 9 | Provide a brief summary of the limitations of the evidence included in the review (e.g. study risk of bias, inconsistency and imprecision). | Yes |
| Interpretation | 10 | Provide a general interpretation of the results and important implications. | Yes |
| **OTHER** |  |  |  |
| Funding | 11 | Specify the primary source of funding for the review. | Yes |
| Registration | 12 | Provide the register name and registration number. | Yes |

*From: Page MJ, McKenzie JE, Bossuyt PM, et al. The PRISMA 2020 statement: an updated guideline for reporting systematic reviews. BMJ 2021; 372: n71. doi: 10.1136/bmj.n71. Distributed under CC BY 4.0.*

**Table S5.** Preferred Reporting Items for Systematic Reviews and Meta-Analyses (PRISMA) 2020 Checklist. Page numbers refer to the revised manuscript (clean version).

| **Section and Topic** | **Item #** | **Checklist item** | **Location** |
| --- | --- | --- | --- |
| **TITLE** |  |  |  |
| Title | 1 | Identify the report as a systematic review. | Page 1 |
| **ABSTRACT** |  |  |  |
| Abstract | 2 | See the PRISMA 2020 for Abstracts checklist. | Pages 2-3 |
| **INTRODUCTION** |  |  |  |
| Rationale | 3 | Describe the rationale for the review in the context of existing knowledge. | Pages 6-7 |
| Objectives | 4 | Provide an explicit statement of the objective(s) or question(s) the review addresses. | Page 7 |
| **METHODS** |  |  |  |
| Eligibility criteria | 5 | Specify the inclusion and exclusion criteria for the review and how studies were grouped for the syntheses. | Page 13 |
| Information sources | 6 | Specify all databases, registers, websites, organisations, reference lists and other sources searched or consulted to identify studies. Specify the date when each source was last searched or consulted. | Page 12 |
| Search strategy | 7 | Present the full search strategies for all databases, registers and websites, including any filters and limits used. | Tables S6-S8 |
| Selection process | 8 | Specify the methods used to decide whether a study met the inclusion criteria of the review, including how many reviewers screened each record and each report retrieved, whether they worked independently, and if applicable, details of automation tools used in the process. | Page 13 |
| Data collection process | 9 | Specify the methods used to collect data from reports, including how many reviewers collected data from each report, whether they worked independently, any processes for obtaining or confirming data from study investigators. | Page 13 |
| Data items | 10a | List and define all outcomes for which data were sought. | Page 13 |
|  | 10b | List and define all other variables for which data were sought. | Page 13 |
| Study risk of bias assessment | 11 | Specify the methods used to assess risk of bias in the included studies. | Page 14 |
| Effect measures | 12 | Specify for each outcome the effect measure(s) used in the synthesis or presentation of results. | Page 14 |
| Synthesis methods | 13a | Describe the processes used to decide which studies were eligible for each synthesis. | Page 14 |
|  | 13b | Describe any methods required to prepare the data for presentation or synthesis. | Page 14 |
|  | 13c | Describe any methods used to tabulate or visually display results of individual studies and syntheses. | Page 14 |
|  | 13d | Describe any methods used to synthesize results and provide a rationale for the choice(s). | Page 14 |
|  | 13e | Describe any methods used to explore possible causes of heterogeneity among study results. | Not applicable |
|  | 13f | Describe any sensitivity analyses conducted to assess robustness of the synthesized results. | Not applicable |
| Reporting bias assessment | 14 | Describe any methods used to assess risk of bias due to missing results in a synthesis. | Page 14 |
| Certainty assessment | 15 | Describe any methods used to assess certainty (or confidence) in the body of evidence for an outcome. | Page 14 |
| **RESULTS** |  |  |  |
| Study selection | 16a | Describe the results of the search and selection process, ideally using a flow diagram. | Page 17; Figure 4 |
|  | 16b | Cite studies that might appear to meet the inclusion criteria, but which were excluded, and explain why. | Page 17 |
| Study characteristics | 17 | Cite each included study and present its characteristics. | Pages 17-21; Tables S9-S12 |
| Risk of bias in studies | 18 | Present assessments of risk of bias for each included study. | Tables S13-S16 |
| Results of individual studies | 19 | For all outcomes, present, for each study, summary statistics and an effect estimate and its precision. | Pages 18-21 |
| Results of syntheses | 20a | For each synthesis, briefly summarise the characteristics and risk of bias among contributing studies. | Pages 17-18 |
|  | 20b | Present results of all statistical syntheses conducted. | Pages 18-21 |
|  | 20c | Present results of all investigations of possible causes of heterogeneity among study results. | Not applicable |
|  | 20d | Present results of all sensitivity analyses conducted. | Not applicable |
| Reporting biases | 21 | Present assessments of risk of bias due to missing results. | Page 20 |
| Certainty of evidence | 22 | Present assessments of certainty in the body of evidence for each outcome assessed. | Page 20 |
| **DISCUSSION** |  |  |  |
| Discussion | 23a | Provide a general interpretation of the results in the context of other evidence. | Pages 22-25 |
|  | 23b | Discuss any limitations of the evidence included in the review. | Page 28 |
|  | 23c | Discuss any limitations of the review processes used. | Pages 26-28 |
|  | 23d | Discuss implications of the results for practice, policy, and future research. | Page 29 |
| **OTHER INFORMATION** |  |  |  |
| Registration and protocol | 24a | Provide registration information for the review, including the register name and registration number. | Page 12 |
|  | 24b | Indicate where the review protocol can be accessed. | Page 12 |
|  | 24c | Describe and explain any amendments to information provided at registration or in the protocol. | Not applicable |
| Support | 25 | Describe sources of financial or non-financial support for the review. | Page 29 |
| Competing interests | 26 | Declare any competing interests of review authors. | Page 29 |
| Availability of data, code and other materials | 27 | Report which of the following are publicly available and where they can be found. | Page 30 |

*From: Page MJ, McKenzie JE, Bossuyt PM, et al. The PRISMA 2020 statement: an updated guideline for reporting systematic reviews. BMJ 2021; 372: n71. Distributed under CC BY 4.0.*

**Table S6.** Full search strategy for Ovid MEDLINE(R) ALL (1946 to 20 November 2025).

| **#** | **Query** | **Results** |
| --- | --- | --- |
| 1 | (semaglutide? or ozempic? or wegovy? or rybelsus?).mp. | 3,842 |
| 2 | exp Glucagon-Like Peptide 1/ae, to [Adverse Effects, Toxicity] | 674 |
| 3 | ((glucagon-like peptide 1 or glucagon like peptide 1 or glp-1 or glp1) adj3 (agonist* or analog* or receptor agonist*)).mp. | 15,464 |
| 4 | 1 or 2 or 3 | 17,199 |
| 5 | exp Anxiety Disorders/ | 97,357 |
| 6 | (panic attack$ or panic disorder or anxiety).mp. | 378,178 |
| 7 | 5 or 6 | 413,279 |
| 8 | 4 and 7 | 108 |

*Search executed 21 November 2025.*

**Table S7.** Full search strategy for Embase Classic and Embase (1947 to 19 November 2025).

| **#** | **Query** | **Results** |
| --- | --- | --- |
| 1 | exp semaglutide/ae, to [Adverse Drug Reaction, Drug Toxicity] | 1,008 |
| 2 | exp glucagon like peptide 1 receptor agonist/ae, to [Adverse Drug Reaction, Drug Toxicity] | 5,640 |
| 3 | (semaglutide? or ozempic? or wegovy? or rybelsus?).mp. | 10,600 |
| 4 | ((glucagon-like peptide 1 or glucagon like peptide 1 or glp-1 or glp1) adj3 (agonist* or analog* or receptor agonist*)).mp. | 31,589 |
| 5 | 1 or 2 or 3 or 4 | 37,935 |
| 6 | exp anxiety disorder/et, si [Etiology, Side Effect] | 21,488 |
| 7 | (panic attack$ or panic disorder or anxiety).mp. | 648,300 |
| 8 | 6 or 7 | 655,789 |
| 9 | 5 and 8 | 552 |

*Search executed 21 November 2025.*

**Table S8.** Full search strategy for the Cochrane Central Register of Controlled Trials (CENTRAL) (inception to 24 November 2025).

| **#** | **Query** | **Results** |
| --- | --- | --- |
| #1 | MeSH descriptor: [Glucagon-Like Peptide 1] explode all trees | 2,549 |
| #2 | semaglutide* OR ozempic* OR wegovy OR rybelsus* | 1,784 |
| #3 | (glucagon-like peptide 1 OR glucagon like peptide 1 OR glp-1 OR glp1) NEAR/3 (agonist* OR analog* OR receptor agonist*) | 17,899 |
| #4 | #1 OR #2 OR #3 | 20,603 |
| #5 | MeSH descriptor: [Anxiety Disorders] explode all trees | 11,064 |
| #6 | MeSH descriptor: [Panic Disorder] explode all trees | 1,117 |
| #7 | (panic attack* OR panic disorder OR anxiety) | 94,445 |
| #8 | #5 OR #6 OR #7 | 96,216 |
| #9 | #4 AND #8 | 496 |

*Search executed 24 November 2025. The search returned 496 records across all Cochrane Library segments. Of these, 342 were trial records and were exported for screening. Cochrane Reviews, Cochrane Protocols and Cochrane Clinical Answers were not eligible publication types and were not exported. No language filter was applied to the search; as prespecified in the registered protocol, studies not published in English were excluded during screening. See PRISMA flow in Figure 4 of the main manuscript.*

**Table S9.** Summary characteristics of clinical trials evaluating semaglutide and panic attacks.

| **Citation & location** | **Study design & duration** | **Population** | **Primary objective** | **Intervention** | **Comparator** | **Outcome & effect size** | **Quality** |
| --- | --- | --- | --- | --- | --- | --- | --- |
| Wadden et al. (2024)^1^ Multinational | Post-hoc analysis of pooled data from four phase 3a randomized, double-blind, placebo-controlled trials (STEP 1, 2, 3 and 5) Duration: >=68 weeks per trial | N = 3,377 Adults >=18 years BMI >=27 kg/m^2^ (with comorbidity) or >=30 kg/m^2^ STEP 2 included type 2 diabetes Mean age 49 years; 69% female; mean BMI 38 Exclusions: severe baseline psychopathology, recent suicidality | To evaluate the psychiatric safety of subcutaneous semaglutide 2.4 mg once weekly versus placebo for chronic weight management | Semaglutide 2.4 mg subcutaneously once weekly (n = 2,116) | Volume-matched placebo subcutaneously once weekly (n = 1,261) | Panic attacks (MedDRA preferred term): Semaglutide 3 (0.14%); placebo 2 (0.16%) Calculated risk ratio 0.89 (95% CI 0.15 to 5.34)^dagger^  Anxiety disorders and symptoms (MedDRA HLGT): Semaglutide 75 (3.5%); placebo 50 (4.0%) Calculated risk ratio 0.89 (95% CI 0.63 to 1.27)^dagger^ | RoB 2: Low risk Underpowered for rare events; exclusion of severe baseline psychopathology limits real-world generalizability |

Abbreviations: BMI, body mass index; CI, confidence interval; HLGT, High-Level Group Term; MedDRA, Medical Dictionary for Regulatory Activities; RoB 2, Risk of Bias 2 tool; STEP, Semaglutide Treatment Effect in People with Obesity. ^dagger^ Effect size calculated by the authors using raw event data from original trial supplementary materials.

**Table S10.** Summary characteristics of observational and cross-sectional studies evaluating semaglutide and acute anxiety.

| **Citation & location** | **Study design & duration** | **Population** | **Intervention** | **Comparator** | **Outcome & effect size** | **Quality** |
| --- | --- | --- | --- | --- | --- | --- |
| De Giorgi et al. (2024)^2^ USA (TriNetX) | Retrospective cohort (1:1 propensity-score matched) 12 months follow-up | N = 46,772 (paired cohort) Mean age 56.6 years; 51.4% female Indication: type 2 diabetes 52.2% with baseline psychiatric disorders | Semaglutide (n = 23,386) | Sitagliptin (active control) (n = 23,386) | Anxiety disorders (ICD-10): Hazard ratio 0.92 (95% CI 0.84 to 1.01) | ROBINS-I: moderate overall risk of bias (Table S14) Retrospective EHR design risks unmeasured residual confounding |
| Kornelius et al. (2024)^3^ USA (TriNetX) | Retrospective cohort (1:1 propensity-score matched) Up to 5 years (Jan 2015 to Dec 2022) | N = 324,506 (162,253 per matched cohort) Mean age 52.4 years; 55.6% female BMI >=30 kg/m^2^ No baseline psychiatric history (excluded by design) | Semaglutide: Wegovy 2.4 mg (n = 30,962) Ozempic 1 mg (n = 76,801) | Obese patients not prescribed GLP-1 RAs (inactive control) (n = 162,253) | Anxiety (ICD-10) for the Wegovy 2.4 mg subgroup: Hazard ratio 2.36 (95% CI 2.23 to 2.49) | ROBINS-I: serious overall risk of bias (Table S14) Inactive comparator introduces severe confounding by indication; relies on accurate ICD coding |
| Do et al. (2025)^4^ USA (Komodo Healthcare Map) | Retrospective cohort (difference-in-differences) 12-month baseline; 12-month follow-up | N = 774,968 Mean age 58.1 years; 52.8% female Indication: type 2 diabetes (44% with overweight or obesity) First GLP-1 RA or DPP-4i prescription Jan 2019 to Mar 2022 | Semaglutide (injectable or oral) (n = 161,364) | Dipeptidyl peptidase-4 inhibitors (active control) (n = 319,102) | Anxiety-related healthcare utilization (ICD-10 F40-F45, F48):Semaglutide-specific incidence rate ratio for office visits 0.81 (95% CI 0.76 to 0.87); emergency department 0.98 (0.85 to 1.12); inpatient 1.26 (0.90 to 1.77); outpatient hospital 0.99 (0.97 to 1.02). Source: Appendix 3, Table 6. | ROBINS-I: moderate overall risk of bias (Table S14) Claims database depends on diagnostic coding accuracy; does not capture mechanisms |
| Balata et al. (2025)^5^ Germany (single-centre hospital outpatient clinic) | Retrospective single-centre analysis 1 year | N = 122 Median age 53 years; 69% male; median BMI 35 Indication: obese heart failure | Semaglutide 1 mg subcutaneously (n = 122) | None (within-group comparison to baseline) | Anxiety (HADS-A score): Median decreased from 5 (baseline) to 3 (1 year) | Modified Downs and Black: 14/28, low methodological quality (Table S15) Lacks distinct non-semaglutide control; small sample size limits power |
| Witaszek et al. (2024)^6^ Poland (web-based survey) | Cross-sectional survey September to October 2023 | N = 1,105 Mean age 38.8 years; 100% female; mean BMI 33.3 Self-reported baseline: 13.8% anxiety; 19.1% depression | Semaglutide (n = 232; 100 anxiety events) | Non-users (no pharmacological obesity treatment) (n = 873; 413 events) | Generalized Anxiety Disorder 7-item scale (GAD-7): Mean difference -1.09 (95% CI -1.98 to -0.20) | Modified Downs and Black: 15/28, low methodological quality (Table S15) Social-media recruitment introduces volunteer bias; self-reported anthropometrics; cross-sectional |
| Alali et al. (2025)^7^ Kuwait (online survey) | Cross-sectional survey February to March 2024 | N = 468 89% female; mean BMI 29.3 Indication: weight loss | Semaglutide (Ozempic or Wegovy) subcutaneously (n = 181; 16 events) | Liraglutide (n = 135; 35 events) or tirzepatide (n = 152) | Self-reported anxiety: RR vs liraglutide 0.38 (95% CI 0.22 to 0.67) RR vs tirzepatide 0.80 (95% CI 0.41 to 1.55) | Modified Downs and Black: 14/28, low methodological quality (Table S15) Volunteer and recall bias; unvalidated self-reported instrument |

Abbreviations: BMI, body mass index; CI, confidence interval; DPP-4i, dipeptidyl peptidase-4 inhibitors; EHR, electronic health record; GAD-7, Generalized Anxiety Disorder 7-item scale; GLP-1 RA, glucagon-like peptide-1 receptor agonist; HADS-A, Hospital Anxiety and Depression Scale (anxiety subscale); ICD-10, International Classification of Diseases, 10th revision; RR, risk ratio.

**Table S11.** Summary characteristics of retrospective pharmacovigilance disproportionality analyses evaluating semaglutide and psychiatric outcomes.

| **Citation & country** | **Database & period** | **Study design & adjustments** | **Outcome & signal statistics** | **Case characteristics & onset** | **Quality & limitations** |
| --- | --- | --- | --- | --- | --- |
| Lu et al. (2025)^8^ China (FAERS data) | FAERS, 2010Q1 to 2024Q1 160,624 GLP-1 RA reports without psychiatric AEs; 25,110 with psychiatric AEs Semaglutide psychiatric reports: 6,112 | Retrospective observational pharmacovigilance disproportionality analysis No adjustment (crude ROR) | Anxiety (MedDRA preferred term): Diabetic population: ROR 1.30 (95% CI 1.12 to 1.50) Weight-control population: ROR 0.49 (95% CI 0.40 to 0.60) | Female 63.2%; age 18-65 years 33.3% 11.8% hospitalized; 1.5% life-threatening or fatal Onset time not reported | Relies on spontaneous reporting; cannot calculate true incidence; underreporting bias; no adjustment for confounding by indication |
| Chen et al. (2024)^9^ China (FAERS data) | FAERS 16,529,887 reports retained after de-duplication; 181,238 involving GLP-1 RAs Semaglutide total AE reports: 17,741 Semaglutide psychiatric AEs: 1,033 | Retrospective observational pharmacovigilance disproportionality analysis No adjustment (crude ROR) | Grouped psychiatric AEs (anxiety, panic attack, stress, nervousness): ROR 1.41 (95% CI 1.23 to 1.63), P<0.001 | Female 65.9%; hospitalized 10.7% Median onset 12 days (IQR 4 to 45.5) | Inherent FAERS limitations (no incidence); no adjustment for indication or other confounders |
| Katranski et al. (2025)^10^ Australia (multi-database) | FAERS (US), CVAROD (Canada), DAEN (Australia) 16,678 total psychiatric reports for GLP-1 RAs Semaglutide psychiatric reports: 4,067 | Retrospective observational pharmacovigilance disproportionality analysis No adjustment (crude ROR) | Panic attack (MedDRA preferred term): FAERS ROR 1.46 (95% CI 1.16 to 1.82) DAEN ROR 3.56 (95% CI 1.14 to 11.12) | Median age 60 years (IQR 54 to 68); female 64.2% Reports from US, Canada, Australia Onset time not reported | Disproportionality cannot establish causality; newer drugs have fewer reports, complicating cross-database comparisons |
| Nishida et al. (2025)^11^ Multinational (>130 countries via VigiBase) | VigiBase (WHO) 2,061,901 reports involving antidiabetic (ATC A10) or anti-obesity (ATC A08) medications Semaglutide psychiatric reports: 7,250 | Active-comparator disproportionality analysis with causal forest model Adjusted for age, sex, WHO region; propensity matching for antidepressant or anxiolytic use | Anxiety disorders and symptoms (HLGT): Adjusted ROR 1.26 (95% CI 1.18 to 1.35) Cases: 1,310 | Age 45-64 years 67%; female 67% Americas 70%; Europe 24% Median onset 7 days (IQR 1 to 33) | Residual confounding by indication remains; subjective nature of psychiatric symptoms; significant regional heterogeneity |

Abbreviations: AE, adverse event; ATC, Anatomical Therapeutic Chemical classification; CI, confidence interval; CVAROD, Canada Vigilance Adverse Reaction Online Database; DAEN, Database of Adverse Event Notifications; FAERS, FDA Adverse Event Reporting System; GLP-1 RA, glucagon-like peptide-1 receptor agonist; HLGT, High-Level Group Term; IQR, interquartile range; MedDRA, Medical Dictionary for Regulatory Activities; ROR, reporting odds ratio; WHO, World Health Organization.

**Table S12.** Summary characteristics of clinical case reports evaluating semaglutide and acute psychiatric events.

| **Citation & study design** | **Patient demographics** | **Intervention** | **ADR onset & presentation** | **Management & resolution** | **Causality & comments** |
| --- | --- | --- | --- | --- | --- |
| Stojkovska et al. (2025)^12^ Clinical case report (Slovenia) | Male, late seventies Indication: type 2 diabetes mellitus | Semaglutide 3 mg orally (Rybelsus) | Onset: 28 days (4 weeks) after initiation Symptoms: severe acute exacerbation of distress, inner restlessness and anxiety; depressive mood; suicidal ideation | Drug discontinued Hospitalized in an acute psychiatric inpatient unit Initiated sertraline and quetiapine Anxiety and insomnia improved within several days; full symptom relief 9 days after management | Naranjo score: 7 (probable adverse drug reaction) Neuropsychological testing one week later formally confirmed depressive and anxiety symptoms Definitive causality limited by single case |

Abbreviations: ADR, adverse drug reaction.

**Table S13.** Risk of bias assessment of included randomized clinical trials using the Cochrane Risk of Bias 2 (RoB 2) tool.

| **Citation** | **Randomization process** | **Deviations from intended intervention** | **Missing outcome data** | **Measurement of outcome** | **Selective reporting** | **Overall risk of bias** |
| --- | --- | --- | --- | --- | --- | --- |
| Wadden et al. (2024)^1^ | Low | Low | Low | Low | Low | Low |

**Table S14.** Risk of bias assessment of included non-randomised comparative cohort studies using the Risk Of Bias In Non-randomised Studies of Interventions (ROBINS-I) tool.

| Study | D1 | D2 | D3 | D4 | D5 | D6 | D7 | Overall |
| --- | --- | --- | --- | --- | --- | --- | --- | --- |
| De Giorgi 2024 | Moderate | Low | Low | Low | Moderate | Moderate | Moderate | Moderate |
| Kornelius 2024 | Serious | Serious | Low | Low | Moderate | Serious | Moderate | Serious |
| Do 2025 | Moderate | Moderate | Low | Moderate | Low | Moderate | Moderate | Moderate |

Domains: D1, bias due to confounding; D2, bias in selection of participants into the study; D3, bias in classification of interventions; D4, bias due to deviations from intended interventions; D5, bias due to missing data; D6, bias in measurement of outcomes; D7, bias in selection of the reported result. A target trial and the comparative effect estimate of interest were specified for each study before assessment. Assessments were made independently by two reviewers (B.M. and M.A.O.), with disagreements resolved by consensus. Overall judgements correspond to the highest risk of bias assigned in any domain. A traffic-light summary is shown in Supplementary Figure S2.

**Table S15.** Methodological quality assessment of the included single-arm before-after study and cross-sectional surveys using the modified Downs and Black checklist.

| **Citation** | **Reporting** | **External validity** | **Internal validity (bias)** | **Internal validity (confounding)** | **Power** | **Score (/28) & overall assessment** |
| --- | --- | --- | --- | --- | --- | --- |
| Witaszek et al. (2024)^6^ | Adequate | Inadequate^b^ | Adequate | Inadequate^c^ | Adequate | 15 (low methodological quality) |
| Alali et al. (2025)^7^ | Adequate | Inadequate^b^ | Inadequate^d^ | Inadequate^c^ | Adequate | 14 (low methodological quality) |
| Balata et al. (2025)^5^ | Adequate | Adequate | Adequate | Inadequate^a^ | Inadequate | 14 (low methodological quality) |

^a^ Study lacked a matched, non-semaglutide control group for comparative baseline adjustment. ^b^ Study recruited participants via social media or online support groups, introducing severe volunteer selection bias. ^c^ Cross-sectional study design is incapable of adjusting for longitudinal confounding variables over time. ^d^ Study relied on an unvalidated, self-generated survey instrument to assess anxiety outcomes.

**Table S16.** Causality assessment of included case reports using the Naranjo Adverse Drug Reaction Probability Scale.

| **Naranjo item** | **Stojkovska et al. (2025)^12^** |
| --- | --- |
| Are there previous conclusive reports on this reaction? | 1 |
| Did the adverse event appear after the suspected drug was given? | 2 |
| Did the adverse reaction improve when the drug was discontinued or a specific antagonist was given? | 1 |
| Did the adverse reaction appear when the drug was readministered? | 0 |
| Are there alternative causes that could have caused the reaction? | 2 |
| Did the reaction reappear when a placebo was given? | 0 |
| Was the drug detected in any body fluid in toxic concentrations? | 0 |
| Was the reaction more severe when the dose was increased, or less severe when the dose was decreased? | 0 |
| Did the patient have a similar reaction to the same or similar drugs in any previous exposure? | 0 |
| Was the adverse event confirmed by any objective evidence? | 1 |
| **Total score** | **7** |

*Naranjo scores correspond to one of four causality categories: definite (>=9), probable (5 to 8), possible (1 to 4) and doubtful (<=0). A score of 7 indicates a probable adverse drug reaction.*

**Figure S1.** Flow diagram of FAERS individual case safety reports included in the active-comparator restricted disproportionality analysis. ICSRs were identified for semaglutide, dulaglutide or empagliflozin from 2018Q1 to 2024Q4, de-duplicated using the OpenFDA case identifier, restricted to reports in which the medication of interest was the primary suspect drug, and cleaned via regular-expression normalization mapped to RxNorm and the Observational Health Data Sciences and Informatics framework. Orlistat and linagliptin were considered as additional comparators but excluded because of insufficient panic attack reports (fewer than three panic attack reports each). Final cohort sizes and panic attack counts are shown.

**Figure S2. Traffic-light summary of ROBINS-I risk of bias judgements for the three included non-randomised comparative cohort studies. Domains are D1, bias due to confounding; D2, bias in selection of participants into the study; D3, bias in classification of interventions; D4, bias due to deviations from intended interventions; D5, bias due to missing data; D6, bias in measurement of outcomes; D7, bias in selection of the reported result. A target trial was specified for each study before assessment. Assessments were made independently by two reviewers (B.M. and M.A.O.), with disagreements resolved by consensus. Domain-level judgements are given in Supplementary Table S14. The single-arm before-after study and the two cross-sectional surveys were not assessed with ROBINS-I because they did not estimate comparative intervention effects within a target-trial framework; these are presented in Supplementary Table S15.**

**References**

1. Wadden TA, Brown GK, Egebjerg C et al. Psychiatric safety of semaglutide for weight management in people without known major psychopathology: post hoc analysis of the STEP 1, 2, 3, and 5 trials. *JAMA Intern Med* 2024; **184**: 1290-1300. https://doi.org/10.1001/jamainternmed.2024.4346

2. De Giorgi R, Koychev I, Adler AI et al. 12-month neurological and psychiatric outcomes of semaglutide use for type 2 diabetes: a propensity-score matched cohort study. *EClinicalMedicine* 2024; **74**: 102726. https://doi.org/10.1016/j.eclinm.2024.102726

3. Kornelius E, Huang JY, Lo SC et al. The risk of depression, anxiety, and suicidal behavior in patients with obesity on glucagon-like peptide-1 receptor agonist therapy. *Sci Rep* 2024; **14**: 24433. https://doi.org/10.1038/s41598-024-75965-2

4. Do D, Lee T, Inneh A et al. Glucagon-like peptide-1 use and healthcare resource utilization for depression and anxiety among adults with type 2 diabetes: 2019 to 2023. *J Behav Health Serv Res* 2025. https://doi.org/10.1007/s11414-025-09950-6

5. Balata M, Sugiura A, Hassan M et al. Impact of semaglutide on health outcomes and mood in obese heart failure patients: a retrospective analysis. *Clin Res Cardiol* 2025. https://doi.org/10.1007/s00392-025-02694-5

6. Witaszek T, Kloda K, Mastalerz-Migas A et al. Association between symptoms of depression and generalised anxiety disorder evaluated through PHQ-9 and GAD-7 and anti-obesity treatment in Polish adult women. *Nutrients* 2024; **16**: 2438. https://doi.org/10.3390/nu16152438

7. Alali S, Al-Otaibi W, Ashodian K et al. A comparison of glucagon-like peptide-1 receptor agonists on weight change, side effects, and quality of life in Kuwait. *Front Nutr* 2025; **12**: 1590379. https://doi.org/10.3389/fnut.2025.1590379

8. Lu W, Wang S, Tang H et al. Neuropsychiatric adverse events associated with glucagon-like peptide-1 receptor agonists: a pharmacovigilance analysis of the FDA Adverse Event Reporting System database. *Eur Psychiatry* 2025; **68**: e20. https://doi.org/10.1192/j.eurpsy.2024.1803

9. Chen W, Cai P, Zou W et al. Psychiatric adverse events associated with GLP-1 receptor agonists: a real-world pharmacovigilance study based on the FDA Adverse Event Reporting System database. *Front Endocrinol* 2024; **15**: 1330936. https://doi.org/10.3389/fendo.2024.1330936

10. Katranski J, Liang S, Morris D et al. Psychiatric adverse events linked to glucagon-like peptide 1 analogues: a disproportionality analysis in American, Canadian and Australian adverse event databases. *Int J Clin Pharm* 2025; **47**: 1739-1747. https://doi.org/10.1007/s11096-025-01943-x

11. Nishida K, Chretien B, Dolladille C et al. Psychiatric and psychological adverse effects associated with dulaglutide, semaglutide, and liraglutide: a VigiBase study. *Clin Nutr* 2025; **51**: 252-265. https://doi.org/10.1016/j.clnu.2025.06.011

12. Stojkovska A, Rus Prelog P, Kokalj Palandacic A. Association of semaglutide use with depressive symptoms and suicidal behavior in a patient with type 2 diabetes: a case report. *J Int Med Res* 2025; **53**: 3000605251349393. https://doi.org/10.1177/03000605251349393
